# Bioactive Constituents of *Melipona quadrifasciata* *anthidioides* Geopropolis with Antifungal Activity against *Sporothrix brasiliensis*

**DOI:** 10.1021/acsomega.6c05995

**Published:** 2026-09-12

**Authors:** Gabriel F. dos Santos, Maria Clara N. Sampaio, Rafael F. dos Santos, Aguida A. de Oliveira, Rosane N. Castro

**Affiliations:** † Departamento de Química Orgânica, Instituto de Química, 67825 Universidade Federal Rural do Rio de Janeiro, BR 465-Km7, Seropédica, Rio de Janeiro 23897-000, Brazil; ‡ Departamento de Microbiologia e Imunologia Veterinária, Instituto de Veterinária, Universidade Federal Rural do Rio de Janeiro, BR 465-Km 7, Seropédica, Rio de Janeiro 23897-000, Brazil

## Abstract

Sporotrichosis caused by *Sporothrix brasiliensis* represents an emerging public health challenge in Brazil, with increasing therapeutic limitations and antifungal resistance. This study investigated Brazilian geopropolis produced by *Melipona quadrifasciata* *anthidioides* as a potential source of antifungal bioactive compounds. Bioassay-guided fractionation of the ethanolic extract led to the isolation of three constituents, identified by NMR, Fourier transform infrared spectroscopy (FTIR)-ATR, and LC-HRMS analyses as kaurenoic acid, cinnamoylgrandifloric acid, and 3*S*(+)-vestitol, the latter two being reported for the first time in this matrix. The dichloromethane fraction exhibited the best antioxidant capacity among the tested fractions (EC_50_ = 200.22 μg mL^–1^). In vitro assays against clinical isolates of *S. brasiliensis* revealed minimum inhibitory concentration (MIC) values ranging from 170.18 to 567.11 μg mL^–1^ for the extract and isolated compounds. Among them, 3*S*(+)-vestitol showed the strongest antifungal activity (MIC = 170.18 μg mL^–1^). These findings demonstrate that *M. quadrifasciata* geopropolis contains bioactive diterpenes and isoflavonoids with significant antifungal activity, highlighting its potential as a source of lead compounds for developing new therapeutic strategies against endemic sporotrichosis.

## Graphical abstract

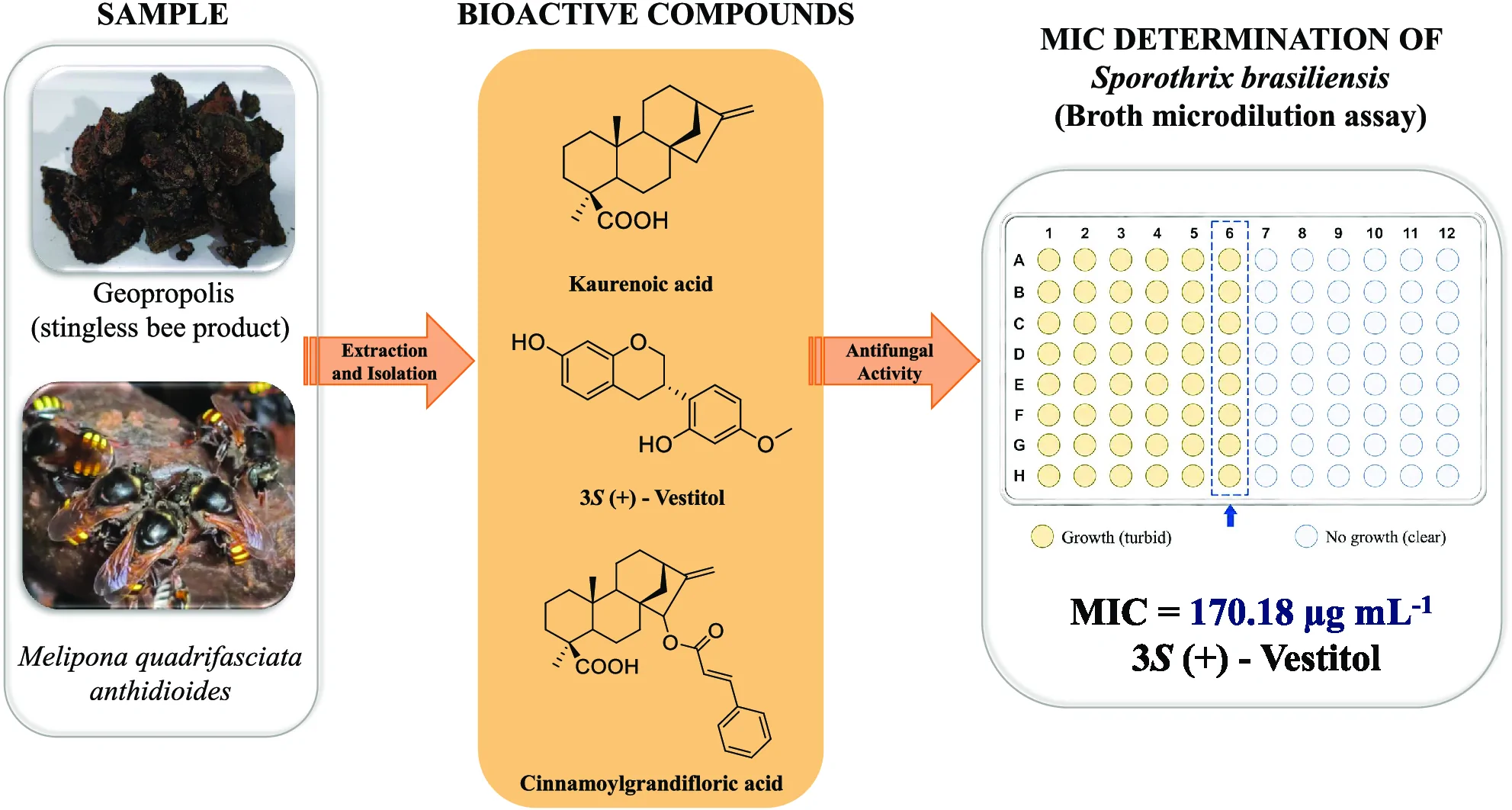

## 1 Introduction

Geopropolis is a resinous material produced by stingless bees through the combination of plant exudates, wax, soil or clay, and salivary secretions.^1^ Bees of the Meliponini tribe comprise more than 600 species distributed across tropical and subtropical regions.^2^ Among them, *M. quadrifasciata* *anthidioides*, popularly known as mandaçaia, is widely distributed in Brazil and plays a significant ecological role in pollination.^3^ The practice of meliponiculture has grown over the years, whether due to beekeepers’ interest in stingless bee species or the commercial potential of the products they generate.^4^ In the hive, geopropolis functions in structural reinforcement, nest sealing, and protection against microbial invasion, reflecting its chemically complex and bioactive composition.^1,5,6^

The chemical composition of geopropolis exhibits a species-specific relationship, wherein variations in the abundance of phenolic compounds, terpenoids, and terpenes are influenced not only by geographic location and collection period but also intrinsically by the producing bee species. Consequently, distinct chemical markers may be observed for different meliponine species.^7^

Chemically, geopropolis is characterized by the presence of fatty acids, organic acids, terpenoids, and a high abundance of phenolic compounds, particularly flavonoids and isoflavonoids.^2,8^ Previous studies have demonstrated anti-inflammatory,^9^ cytotoxic,^10^ antioxidant,^11^ and antimicrobial activities^12^ of stingless bee geopropolis. However, the biological properties of geopropolis vary according to botanical origin, and species of the genus *Melipona* are known to concentrate specialized secondary metabolites often absent in propolis from *Apis mellifera*.^13^

Despite the recognized antimicrobial potential of geopropolis, its activity against *S. brasiliensis* has been scarcely investigated. A recent study by Dos Santos et al.^14^ reported that crude and its fractions of *Melipona mondury* geopropolis extract inhibit *S.* *brasiliensis* in vitro, highlighting the antifungal promise of these resins. However, for *M. quadrifasciata* *anthidioides*, such investigations are virtually absent, and no bioassay-guided isolation studies have been conducted to date. This represents a significant gap in research, since this highly contaminating species requires specific therapies, different from those used for common dermatophytes.^15^

*S. brasiliensis* is the main etiological agent of sporotrichosis in Brazil, where it has caused an expanding zoonotic epidemic primarily associated with infected domestic cats.^16^ Clinical manifestations range from localized cutaneous lesions to systemic infections.^17^ Current treatments rely on itraconazole, fluconazole, and amphotericin B, but therapeutic limitations include prolonged treatment regimens, adverse effects, and emerging antifungal resistance.^18^

Given the urgent demand for new antifungal agents, this study aimed to characterize the phenolic composition and antioxidant capacity of geopropolis from *M. quadrifasciata* *anthidioides* based on its ethanolic extract and its fractions, and to evaluate its in vitro antifungal activity of these preparations against clinical isolates of *S. brasiliensis*, seeking to identify bioactive constituents associated with their antifungal potential.

## 2 Results and Discussion

### 2.1 Ethanol Extract of Geopropolis and Fractionation Profile

To obtain bioactive constituents potentially responsible for the antifungal activity, powdered geopropolis (10 g) was subjected to ultrasonic-assisted extraction (UAE), yielding 1.97 g of ethanolic extract (EEGM), corresponding to 19.75% (w/w). UAE has become an important technique in natural product chemistry due to its efficiency, reduced extraction time, and improved mass transfer, while also aligning with green chemistry principles.^19,20^

Although the extraction yield obtained by UAE (19.75%, w/w) was higher than several values reported for geopropolis extracted by conventional methods,^21^ this comparison should be interpreted cautiously because differences in sample origin, extraction conditions, and moisture content may also contribute to variations in extraction yield. For instance, Bankova et al. reported yields of 5% for *Melipona compressipes* geopropolis and 17% for *M. quadrifasciata* *anthidioides* samples obtained by maceration.^22^ Similarly, Santos et al. reported a 4.8% yield using dynamic maceration.^23^ The higher yield observed in the present study may reflect both the efficiency of UAE and differences in geographical origin, which is known to influence geopropolis chemical composition.

Liquid–liquid partitioning of 750.2 mg of EEGM afforded fractions of increasing polarity. The hexane fraction presented the highest yield (419.30 mg; 55.89 ± 3.11%), followed by the dichloromethane fraction (256.30 mg; 34.16 ± 2.16%), whereas the methanol:water fraction showed a low yield (12.50 mg; 1.66 ± 0.07%). It is important to note that the low yield reflects the residual nature of this fraction after exhaustive sequential liquid–liquid partitioning, rather than an intrinsic inefficiency of hydroalcoholic extraction, as most extractable constituents had already been distributed into the organic phases.

This distribution indicates a predominance of nonpolar and medium-polarity constituents in *M. quadrifasciata* *anthidioides* geopropolis, consistent with the typical occurrence of diterpenes and flavonoids previously reported in stingless bee geopropolis.^1^

### 2.2 Phenolic Content and Radical Scavenging Activity

Following ethanolic extraction of *M. quadrifasciata* geopropolis and subsequent solvent partitioning based on increasing polarity, the crude extract (EEGM) and its hexane (Hex), dichloromethane (DCM), and MeOH:H_2_O fractions were evaluated for total phenolic content (TPC), total flavonoid content (TFC), and antioxidant capacity using the DPPH free radical scavenging activity assay (Table 1).

**Table 1:** Total Phenolic Content (TPC), Total Flavonoid Content (TFC), and Antioxidant Capacity Determined by the DPPH Assay for the Crude Ethanolic Extract (EEGM) and Its Fractions*

Samples	TPC (mg GAE 100 mg^–1^)	TFC (mg QE 100 mg^–1^)	DPPH (EC_50_ μg mL^‑1^)
EEGM	0.63 ± 0.017^a^	0.15 ± 0.003^a^	203.50 ± 0.90^a^
Fr. Hex	ND	ND	ND
Fr. DCM	1.89 ± 0.022^b^	0.42 ± 0.006^b^	200.22 ± 0.70^a^
Fr. MeOH:H_2_O	0.67 ± 0.003^a^	0.23 ± 0.009^a^	467.92 ± 1.13^b^

*Values are expressed as mean ± SD (*n* = 3). Different superscript letters within the same column indicate statistically significant differences (*p* < 0.05). ND = not detected (below the limit of detection).

The DCM fraction exhibited the highest concentrations of phenolic and flavonoid compounds, with values of 1.89 mg GAE 100 mg^–1^ and 0.42 mg QE 100 mg^–1^, respectively. In contrast, the hexane fraction showed no detectable levels of these compounds, while the MeOH:H_2_O fraction and the crude EEGM extract presented intermediate and comparable values. This distribution indicates that liquid–liquid partitioning effectively enriched semi-polar phenolic constituents in the DCM fraction, whereas predominantly nonpolar metabolites were preferentially extracted into the hexane phase. Such enrichment is consistent with the expected polarity of isoflavonoids and related phenolic derivatives later identified in this study.^1^

The TPC and TFC values obtained for the DCM fraction exceed those reported by Hochheim et al.^24^ for *M. quadrifasciata* geopropolis (0.81 mg GAE 100 mg^–1^ and 0.22 mg QE 100 mg^–1^, respectively). Similarly, Cardozo et al.^25^ reported TPC values ranging from 0.29 to 1.11 mg GAE 100 mg^–1^ and TFC values between 0.02 and 0.17 mg QE 100 mg^–1^ for different mandaçaia geopropolis extracts, which are more consistent with the values observed for the crude EEGM in the present study. These comparisons reinforce the effectiveness of the fractionation strategy in concentrating bioactive phenolic constituents, particularly in the DCM fraction.

Discrepancies in extraction yield and metabolite concentration among studies can be attributed primarily to extrinsic factors. As reported by Zin et al.,^26^ extraction methodology - such as sonication, maceration, or Soxhlet extraction - plays a decisive role in secondary metabolite recovery due to differences in solvent penetration, temperature, and extraction time. Moreover, the geographical origin and seasonality strongly influence geopropolis composition, since the diversity of botanical resin sources exploited by stingless bees varies spatially and temporally.^27^ Consequently, ecological variability leads to distinct chemical fingerprints even within the same bee species.

Regarding antioxidant activity, EC_50_ values ranged from 200.22 to 467.92 μg mL^–1^. The DCM fraction displayed the highest radical scavenging capacity (EC_50_ = 200.22 μg mL^–1^), followed closely by the crude ethanolic extract (EC_50_ = 203.50 μg mL^–1^), whereas no detectable antioxidant capacity was observed for the hexane fraction. The close correlation between higher TPC/TFC values and lower EC_50_ supports a direct relationship between phenolic content and antioxidant potential, parameters widely recognized as important indicators of geopropolis quality.^28^

The antioxidant activity of phenolic compounds is largely attributed to their capacity to act as hydrogen or electron donors, neutralizing free radicals and limiting reactive oxygen species (ROS) formation.^29^ Although antioxidant capacity does not directly predict antifungal efficacy, phenolic-rich fractions are frequently associated with redox-modulating and membrane-interacting effects^29^ that may enhance antifungal mechanisms. In this context, the enrichment of semi-polar phenolic constituents in the DCM fraction is noteworthy, as this fraction also demonstrated relevant biological activity in subsequent antifungal assays, suggesting that phenolic metabolites—alone or in synergy with diterpenes—may contribute to the observed inhibition of fungal growth.

Gabriel et al.^30^ reported markedly stronger antioxidant capacity for ethanolic extracts of *M. quadrifasciata* geopropolis collected in Inconfidentes (MG) and Jaguariúna (SP), with EC_50_ values of 6.94 ± 0.09 μg mL^–1^ and 6.78 ± 0.09 μg mL^–1^, respectively. In contrast, geopropolis from Betim (MG) exhibited weaker activity (EC_50_ = 385.0 μg mL^–1^), lower than that of the EEGM extract analyzed herein. Likewise, Bonamigo et al.^31^ reported a DPPH scavenging activity of 60.91 μg mL^–1^ for *M. quadrifasciata* *anthidioides* geopropolis from central-western Brazil, indicating stronger antioxidant potential than that observed in the present study. Such variability further underscores the influence of environmental and botanical factors on chemical composition and, consequently, biological activity.

Taken together, these findings demonstrate that solvent partitioning effectively concentrates semi-polar phenolic constituents in Mandaçaia geopropolis, directly influencing antioxidant capacity and potentially contributing to antifungal activity. The integrated chemical and biological profile observed herein highlights the relevance of phenolic and flavonoid enrichment in the search for bioactive metabolites from geopropolis.

### 2.3 Isolation and Characterization of Compounds from EEGM

Bioassay-guided fractionation of the crude ethanolic extract of *M. quadrifasciata* geopropolis (EEGM) led to the isolation of three major constituents. Compounds **1** and **2** were obtained from the hexane fraction (Fr. Hex) through an antifungal activity-guided approach (see subsequent section), which was prioritized due to both its biological activity and comparatively higher yield. In contrast, compound **3** was isolated from the dichloromethane fraction (Fr. DCM) following an antioxidant-guided strategy, as this fraction exhibited the highest radical scavenging activity. The chemical structures of compounds 1–3 are presented in Figure 1.

**Figure 1:**
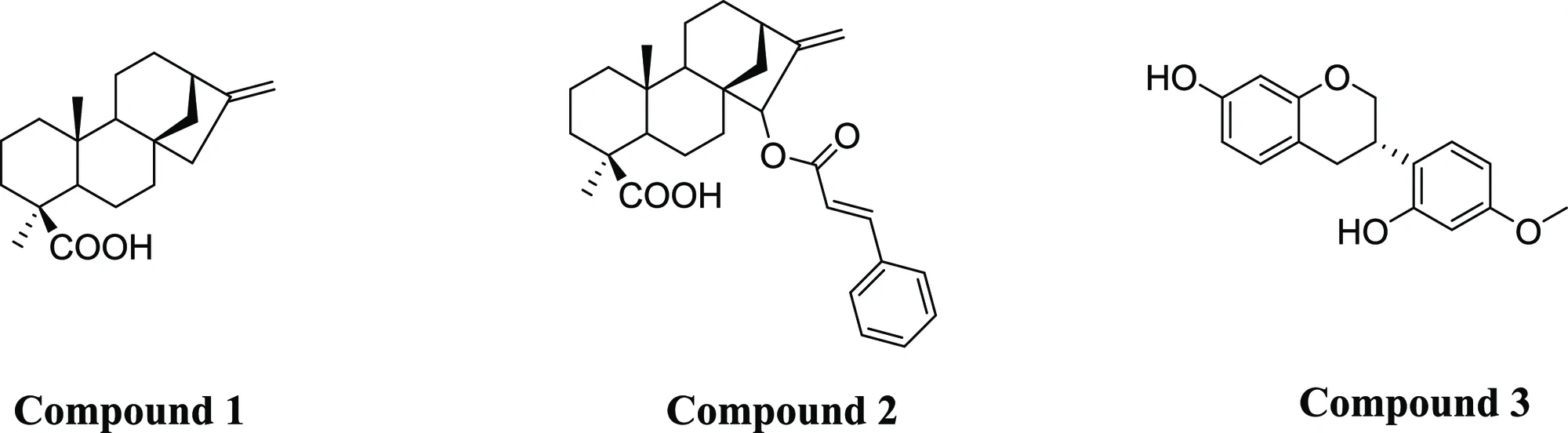
Isolated compounds from *M. quadrifasciata* *anthidioides* Geopropolis. Compound **1**: Kaurenoic acid; compound **2**: Cinnamoylgrandifloric acid; compound **3**: 3*S*(+)-Vestitol.

Compound **1** was obtained as a white amorphous solid and identified as kaurenoic acid. The melting point (162 °C) was consistent with that reported by Monte et al.^32^ HPLC-DAD analysis indicated a purity of 99.31% (Supporting Information Figure S1). The specific optical rotation, [α]_D_^25^ −108.2° (*c* 1, CHCl_3_), was in close agreement with the literature value ([α]_^D^_^25^ −107.3°, *c* 0.31, CHCl_3_) reported by Hsieh et al.^33^

The FTIR spectrum displayed a strong absorption band at 2880 cm^–1^, attributed to sp^3^ C–H stretching vibrations (methyl and methylene groups). A broad absorption in the 3000–2500 cm^–1^ region indicated O–H stretching of a carboxylic acid, supported by a carbonyl stretching band at 1690 cm^–1^. An additional band at 1660 cm^–1^ was assigned to the C=C stretching vibration of an exocyclic double bond in a five-membered ring (Supporting Information Figure S2).

The ^1^H NMR spectrum (Supporting Information Figure S3) showed two singlets at *δ*_H_ 0.89 and 1.11 (each 3H, s), corresponding to methyl groups attached to quaternary carbons. Signals at *δ*_H_ 4.60 and 4.40 (each 1H, s) were assigned to the non-equivalent geminal protons of an exocyclic double bond. The ^13^C NMR spectrum (Supporting Information Figure S4) exhibited a signal at *δ*_C_ 179.06 (carboxylic acid carbonyl), *δ*_C_ 155.48 (quaternary olefinic carbon), and *δ*_C_ 103.81 (terminal methylene carbon), consistent with a kaurane-type diterpenoid skeleton.^34^ Full ^1^H and ^13^C NMR spectroscopic assignments are available in the Supporting Information Table S1.

With the aid of HMBC (Heteronuclear Multiple Bond Correlation) experiments, the long-range couplings were established, and the carbon chemical shifts were fully assigned. A ^3^ *J*_CH_ coupling was observed between the carboxyl carbon C-19 (*δ*_C_ 179.06) and the methyl protons at C-18 (*δ*_H_ 1.10) (as illustrated in Supporting Information Figure S5). Additionally, a ^3^ *J*_CH_ correlation was detected between the exocyclic double bond quaternary carbon C-16 (*δ*_C_ 155.4) and the methylene protons at C-15 (*δ*_H_ 1.99). Furthermore, ^3^ *J*_CH_ couplings were observed between the vinylic protons of the exocyclic methylene group 2H-17 and both C-15 (*δ*_C_ 48.70) and C-13 (*δ*_C_ 43.60). These correlations allowed for the unambiguous determination of the methyl group adjacent to the carboxylic acid moiety, as well as the definitive confirmation of the exocyclic double bond position.

High-resolution mass spectrometry HRMS, ESI negative mode; (Supporting Information Figure S6) revealed a deprotonated molecular ion [M–H]^‑^ at *m*/*z* 301.2182, in excellent agreement with the calculated value for C_20_H_29_O_2_^–^ (301.2173), thereby confirming the identity of compound **1** as kaurenoic acid.

The occurrence of kaurenoic acid in geopropolis is consistent with previous reports on stingless bee products. Velikova et al.^35^ isolated kaurenoic acid and related *ent*-kaurene diterpenes from *M. quadrifasciata* *anthidioides* geopropolis collected in Paraná, Brazil, associating these compounds with antibacterial activity.

Compound **2** was isolated as a white solid and identified as cinnamoylgrandifloric acid (kaur-16-en-19-oic acid, 15-[(1-oxo-3-phenyl-2-propen-1-yl)­oxy]). The melting point (190 °C) agreed with that reported by Bertolucci et al.^36^ HPLC-DAD analysis indicated a purity of 96.53% (Supporting Information Figure S7).

The FTIR spectrum showed characteristic bands of a kaurene skeleton, including sp^3^ C–H stretching at 2880 cm^–1^, a broad O–H stretching band (3000–2500 cm^–1^), and a carboxylic acid carbonyl stretch at 1690 cm^–1^. Additionally, an absorption at approximately 1715 cm^–1^, characteristic of an *α,β*-unsaturated ester carbonyl, together with a C–O stretching band at 1169 cm^–1^ and a C=C stretching band at 1640 cm^–1^, confirmed the presence of an esterified cinnamoyl moiety (Supporting Information Figure S8).

The ^1^H NMR spectrum (Supporting Information Figure S9) revealed resonances typical of the kaurene framework along with diagnostic signals for the cinnamoyl group. Doublets at *δ*_H_ 7.65 and 6.66 (each 1H, d, *J* = 16.0 Hz) indicated a *trans α,β*-unsaturated system, while multiplets between *δ*_H_ 7.43–7.55 (5H, m) corresponded to a monosubstituted aromatic ring. The ^13^C NMR spectrum (Supporting Information Figure S10) showed a signal at *δ*_C_ 166.60 (C, ester carbonyl) and signals at *δ*_C_ 145.12 (CH) and 118.68 (CH) assigned to the *α,β*-unsaturated carbons, further supporting the cinnamate ester structure. Full ^1^H and ^13^C NMR spectroscopic assignments are available in the Supporting Information Table S2.

HMBC analysis further confirmed that the cinnamate ester moiety is attached at the C-15 position, as evidenced by a ^3^ *J*_CH_ correlation observed between the ester carbonyl carbon C-9′ (*δ*_C_ 166.60) and the proton at C-15 (*δ*_H_ 5.29). Moreover, the experiment enabled the full assignment of chemical shifts within the cinnamic portion of the molecule, thereby corroborating the presence of this structural unit. The key long-range correlations observed for the cinnamate moiety were as follows: the quaternary carbonyl carbon C-9′ (*δ*_C_ 166.60) exhibited a ^3^ *J*_CH_ coupling with H-7′ (*δ*_H_ 7.65) and a ^2^ *J*_CH_ coupling with H-8′ (*δ*_H_ 6.66); the olefinic carbon C-7′ (*δ*_C_ 145.12) showed a ^3^ *J*_CH_ coupling with the aromatic protons H-2′/6′ (*δ*_H_ 7.75); the aromatic quaternary carbon C-1′ (*δ*_C_ 135.0) displayed ^3^ *J*_CH_ correlations with H-8′ (*δ*_H_ 6.66) and H-3′/5′ (*δ*_H_ 7.43); and the olefinic carbon C-8′ (*δ*_C_ 118.68) showed a ^2^ *J*_CH_ coupling with H-7′ (*δ*_H_ 7.65) (Supporting Information Figure S11).

HRMS analysis (ESI negative mode; Supporting Information Figure S12) displayed a deprotonated molecular ion [M–H]^‑^ at *m*/*z* 447.2545, matching the calculated value for C_29_H_35_O_4_^–^ (447.2541). Together, these data unequivocally established compound **2** as cinnamoylgrandifloric acid.

The detection of cinnamoylgrandifloric acid in geopropolis is particularly noteworthy, representing the first report of this compound in a *Melipona* geopropolis matrix. Although frequently isolated from plants of the genus *Mikania*,^37^ its presence in geopropolis suggests selective resin foraging behavior and highlights the chemical diversity resulting from bee–plant interactions.

Compound **3** was obtained as a white-beige solid and identified as 3*S*(+)-vestitol, with a specific optical rotation of [α]^25_D_^ +13.6° (*c* 1, MeOH). HPLC-DAD analysis indicated a purity of approximately 95.19% (Supporting Information Figure S13).

The FTIR spectrum exhibited a broad O–H stretching band at 3400 cm^–1^ and a C–O stretching band near 1100 cm^–1^. Aromatic C=C stretching vibrations were observed in the 1600–1610 cm^–1^ region, consistent with a phenolic isoflavan structure (Supporting Information Figure S14).

The ^1^H and ^13^C NMR data (Supporting Information Figure S15 and Figure S16) were in full agreement with literature values for vestitol.^38^ The ^1^H NMR spectrum showed aromatic proton signals between *δ*_H_ 6.38 and 7.04, corresponding to the A- and B-rings of the isoflavan skeleton. Signals at *δ*_H_ 4.36 (1H, br d) and 4.06 (1H, t, 10.2 Hz) were assigned to the diastereotopic protons at C-2 (equatorial and axial, respectively), while a singlet at *δ*_H_ 3.79 (3H, s) corresponded to a methoxyl group and a signal at *δ*_H_ 3.52 (1H, m) to the chiral proton at C-3. The ^13^C NMR spectrum confirmed the isoflavan framework, with characteristic signals at *δ*_C_ 69.9 (C-2, deshielded due to oxygen attachment), *δ*_C_ 31.69 (chiral methine carbon, C-3), and *δ*_C_ 30.34 (methylene carbon, C-4). Full ^1^H and ^13^C NMR spectroscopic assignments are available in the Supporting Information Table S3.

Structural confirmation was further supported by LC–MS analysis (ESI negative mode; Supporting Information Figure S17), which showed the deprotonated molecular ion [M–H]^‑^ at *m*/*z* 271, consistent with the molecular formula C_16_H_15_O_4_^–^.

To the best of our knowledge, this is the first report of 3*S*(+)-vestitol isolated from *M. quadrifasciata* geopropolis. Although vestitol is widely recognized as a chemical marker of Brazilian red propolis,^39^ its occurrence in geopropolis underscores the chemical convergence among bee products and reinforces the relevance of stingless bee geopropolis as a source of bioactive phenolic constituents.

### 2.4 Antifungal Activity Evaluation of EEGM, Fractions, and Isolated Compounds against *S. brasiliensis*

The antifungal activity of the ethanolic extract of mandaçaia geopropolis (EEGM), its liquid–liquid partition fractions (Hex, DCM, and MeOH:H_2_O), and the isolated compounds—kaurenoic acid, cinnamoylgrandifloric acid, and 3*S*(+)-vestitol—was evaluated against a drug-resistant clinical isolate of *S. brasiliensis* using the broth microdilution method. Minimum inhibitory concentration (MIC) values are summarized in Table 2 and compared with the reference antifungal agent itraconazole, the first-line therapy for sporotrichosis.

**Table 2:** Antifungal Activity (MIC, μg mL^-1^) of EEGM, Fractions, and Isolated Compounds against *S. brasiliensis* ^a^

Samples	MIC (μg mL^‑1^)
EEGM	562.50
Fr. Hex	281.25
Fr. DCM	562.50
Fr. MeOH:H_2_O	562.50
kaurenoic acid	567.11
cinnamoylgrandifloric acid	210.39
3*S* (+)-vestitol	170.18
Itraconazole	> 16.00

a All assays were performed duplicate and independently repeated on two different days, yielding identical and concordant MIC values; therefore, no standard deviation is reported (*n* = 2 per experiment).

All samples exhibited measurable antifungal activity against *S. brasiliensis*, with MIC values ranging from 170.18 to 567.11 μg mL^–1^. In contrast, itraconazole did not inhibit fungal growth at the highest concentration tested (MIC > 16 μg mL^–1^), confirming the resistant phenotype of the clinical isolate and underscoring the urgent need for alternative antifungal agents.

Among the fractions, the hexane fraction (Fr. Hex) demonstrated the strongest antifungal activity (MIC = 281.25 μg mL^–1^), suggesting that lipophilic constituents substantially contribute to the observed effect. This finding directly guided the bioassay-driven isolation strategy, leading to the identification of kaurenoic acid (MIC = 567.11 μg mL^–1^) and cinnamoylgrandifloric acid (MIC = 210.39 μg mL^–1^). Notably, cinnamoylgrandifloric acid exhibited significantly greater potency than kaurenoic acid, indicating that the antifungal activity of the hexane fraction is primarily attributable to this compound. The enhanced activity may be associated with the presence of the cinnamoyl moiety, which could increase lipophilicity and promote interactions with fungal membranes. Terpenoids are widely reported to exert antifungal effects through interactions with membrane lipids, leading to membrane destabilization and increased permeability.^40^

Among the isolated compounds, 3*S*(+)-vestitol displayed the lowest MIC value (170.18 μg mL^–1^), representing the most active compound tested. Interestingly, despite this potency, the DCM fraction itself did not exhibit superior antifungal activity. This discrepancy suggests the occurrence of antagonistic or dilution effects within the complex fraction matrix, where inactive or weakly active constituents may reduce the effective concentration of vestitol. The pronounced activity of vestitol may be related to its isoflavonoid scaffold, as flavonoids are known to impair fungal viability through multiple mechanisms, including mitochondrial dysfunction via inhibition of the electron transport chain, efflux pump inhibition, and interference with cell wall biosynthesis and cell division.^41^

To the best of our knowledge, this is the first report describing the antifungal activity of kaurenoic acid, cinnamoylgrandifloric acid, and 3*S*(+)-vestitol against clinical isolates of *S. brasiliensis*. These findings are particularly relevant in the context of sporotrichosis, a neglected mycosis of increasing public health concern in Brazil. Although the antifungal activity of kaurenoic acid has been previously reported against other fungal species, including *Penicillium notatum*, *Microsporum gypseum*, and *Epidermophyton floccosum*,^42^ its activity against *S. brasiliensis* had not been documented prior to this study.

Similarly, vestitol has been reported as an antifungal agent against several pathogenic fungi. Gordon et al.^43^ described MIC values of 100 μg mL^–1^ against *Cryptococcus neoformans* and *Aspergillus fumigatus*, 50 μg mL^–1^ against *Histoplasma capsulatum*, and 25 μg mL^–1^ against *Sporothrix schenckii*. Although vestitol showed comparatively lower potency in the present study, this difference may reflect species-specific susceptibility, as well as the resistant phenotype of the *S. brasiliensis* clinical isolate evaluated.

Few studies have investigated the antifungal activity of geopropolis against *S. brasiliensis*. Waller et al.^16^ evaluated Brazilian green propolis from *A. mellifera* and propolis from *Tetragonisca angustula* at concentrations ranging from 50 to 1.56 mg mL^–1^ and reported no antifungal activity. In contrast, all extracts, fractions, and isolated compounds from mandaçaia geopropolis evaluated herein exhibited measurable inhibitory effects. The crude extract and its fractions MIC values reported by Dos Santos et al.^14^ for *M. mondury* geopropolis (281.25–562.50 μg mL^–1^) are consistent with our findings. Critically, however, the present study goes further by isolating and testing purified constituents, revealing that cinnamoylgrandifloric acid (MIC = 210.39 μg mL^–1^) and 3*S* (+)-vestitol (MIC = 170.18 μg mL^–1^) are the principal antifungal agents. Collectively, these results highlight the distinctive chemical profile of mandaçaia geopropolis and reinforce its potential as a source of bioactive metabolites against drug-resistant *Sporothrix* species. The identification of cinnamoylgrandifloric acid and 3*S*(+)-vestitol as the most active constituents provides a rational basis for further mechanistic studies and in vivo evaluation, supporting the development of alternative therapeutic strategies for refractory sporotrichosis.

These findings should be interpreted as an initial pharmacological indication, and further studies including structure–activity relationship investigations and in vivo assays are necessary to fully assess the therapeutic potential of these compounds.

Although the MIC values observed in this study can be considered moderate, their relevance should be interpreted in the context of the drug-resistant clinical isolate employed, for which the reference antifungal itraconazole showed no inhibitory activity. In this scenario, the detected antifungal effects highlight the potential of these natural products as preliminary leads for further optimization rather than as immediately potent antifungal agents. These findings should be interpreted as an initial pharmacological indication, and further studies including structure–activity relationship investigations and in vivo assays are necessary to fully assess the therapeutic potential of these compounds.

## 3 Conclusion

This study presents a comprehensive chemical and biological investigation of mandaçaia geopropolis produced by *M. quadrifasciata* *anthidioides*. The total phenolic and flavonoid contents were consistent with previously reported values for geopropolis from this stingless bee species, supporting the observed antioxidant capacity.

Bioassay-guided fractionation coupled with spectroscopic analyses led to the identification of kaurenoic acid, cinnamoylgrandifloric acid, and the isoflavonoid 3*S*(+)-vestitol. Notably, cinnamoylgrandifloric acid and 3*S*(+)-vestitol are reported herein for the first time in mandaçaia geopropolis, thereby expanding the current knowledge of its chemical diversity.

The antifungal assays demonstrated that the crude extract, its fractions, and the isolated compounds inhibit the growth of *S. brasiliensis*, with 3*S*(+)-vestitol exhibiting the most pronounced activity against this pathogen. These findings establish the activity of these metabolites against *S. brasiliensis* for the first time and underscore their potential relevance in the context of sporotrichosis. Future perspectives include mechanistic investigations and the exploration of these natural products as promising scaffolds for the development of novel antifungal agents.

Despite the moderate antifungal activity observed, the results provide a relevant basis for future optimization and reinforce the potential of geopropolis-derived metabolites as promising scaffolds in antifungal drug discovery.

## 4 Experimental Section

### 4.1 General Procedures

All solvents and reagents used for spectrophotometric and chromatographic analyses were of spectroscopic grade (VETEC, Rio de Janeiro, Brazil). Ultrapure water was obtained from a Milli-Q purification system (Millipore, São Paulo, Brazil). The standards 2,2-diphenyl-1-picrylhydrazyl (DPPH) and Folin–Ciocalteu reagent were obtained from Sigma-Aldrich (Merck).

IR spectra were recorded on a Bruker Vertex 70 spectrophotometer equipped with an ATR accessory. UV–Vis analyses were performed using a Bel Photonics spectrophotometer and a Bio-Rad microplate reader.

NMR spectra (^1^H and ^13^C, 1D and 2D) were acquired on a Bruker Avance III 500 MHz spectrometer (500 MHz for ^1^H and 125 MHz for ^13^C). CDCl_3_ and DMSO-*d*_6_ were used as solvents, with tetramethylsilane (TMS) as internal standard. Chemical shifts (*δ*) are reported in ppm and coupling constants (*J*) in Hz.

High-resolution mass spectra were obtained using an Agilent 1290 Infinity II LC system coupled to an Agilent 6530 Q-TOF mass spectrometer (Agilent Technologies, Santa Clara, CA) equipped with a Zorbax Extend C18 column (50 × 2.1 mm^2^, 1.8 μm). The column temperature was maintained at 40 °C, injection volume was 10 μL, and flow rate was 0.50 mL min^–1^. The mobile phase consisted of water with 0.1% formic acid and 5 mM ammonium formate (A) and acetonitrile with 0.1% formic acid and 5 mM ammonium formate (B). The gradient program was: 50% B (0–5 min), 50–95% B (5–8 min), 95–100% B (8–9.5 min), 100% B (9.5–11 min), followed by re-equilibration to initial conditions. Mass spectrometric detection was performed using electrospray ionization (ESI) in positive or negative mode. Operating parameters were: nitrogen gas flow, 12 L min^–1^; gas temperature, 300 °C; nebulizer pressure, 38 psi; capillary voltage, 3500 V; fragmentor voltage, 120 V. Full-scan data were acquired over *m*/*z* 50–3200. Data acquisition and processing were performed using MassHunter software (v11.0).

The EEGM extract and its fractions were also qualitatively analyzed by high-performance liquid chromatography with diode array detection (HPLC-DAD) at a concentration of 5 mg/mL (Supporting Information Figure S18). The chromatographic profiling was performed on a Prominence-Shimadzu liquid chromatograph equipped with two LC-20AT pumps, an SPD-M20A DAD detector, and an SIL-10A autosampler. System control and data acquisition were carried out using LCSolution software (Shimadzu). Separations were conducted on a reversed-phase C18 analytical column (250 × 4.6 mm^2^, 5 μm particle size, Betasil, Thermo) maintained at 40 °C. The mobile phase consisted of water with 1% acetic acid (solvent A) and methanol with 1% acetic acid (solvent B), at a constant flow rate of 1.0 mL/min. The elution gradient was programmed as follows: starting at 35% B, increasing to 75% B over 10 min, then to 80% B over 15 min, then to 95% B over 18 min, followed by a decrease to 70% B over 20 min, and finally returning to 35% B over 2 min, which was held for an additional 2 min. Chromatograms were monitored at 280 nm, as most geopropolis constituents exhibit their maximum UV absorption near this wavelength.

### 4.2 Geopropolis Extraction

Geopropolis from *M. quadrifasciata* *anthidioides* was obtained from beekeepers in Bosque da Barra, Rio de Janeiro, Brazil (22°59′ S, 43°22′ W). The material was stored frozen and ground to a fine powder. Powdered geopropolis (10 g) was extracted 3 times with ethanol (95%, 200 mL each) for 2 h in an ultrasonic bath at room temperature (40 kHz, UltraCleane 1600, UNIQUE). After filtration to remove inorganic residues, the combined extracts were concentrated under reduced pressure at 40 °C to yield the ethanolic extract (EEGM, 1.97 g).

### 4.3 Fractionation of Ethanolic Extract

The overall fractionation and isolation workflow is depicted in Scheme 1. EEGM (750.2 mg) was dissolved in MeOH/H_2_O (7:3, v/v) and sequentially partitioned with hexane (4 × 50 mL) followed by dichloromethane (4 × 50 mL). Organic layers were dried over anhydrous Na_2_SO_4_, filtered, and concentrated under reduced pressure at 40 °C to afford: hexane fraction (Fr. Hex, 419.3 mg), dichloromethane fraction (Fr. DCM, 256.3 mg), and hydroalcoholic fraction (Fr. MeOH/H_2_O, 12.5 mg).

**Scheme 1:**
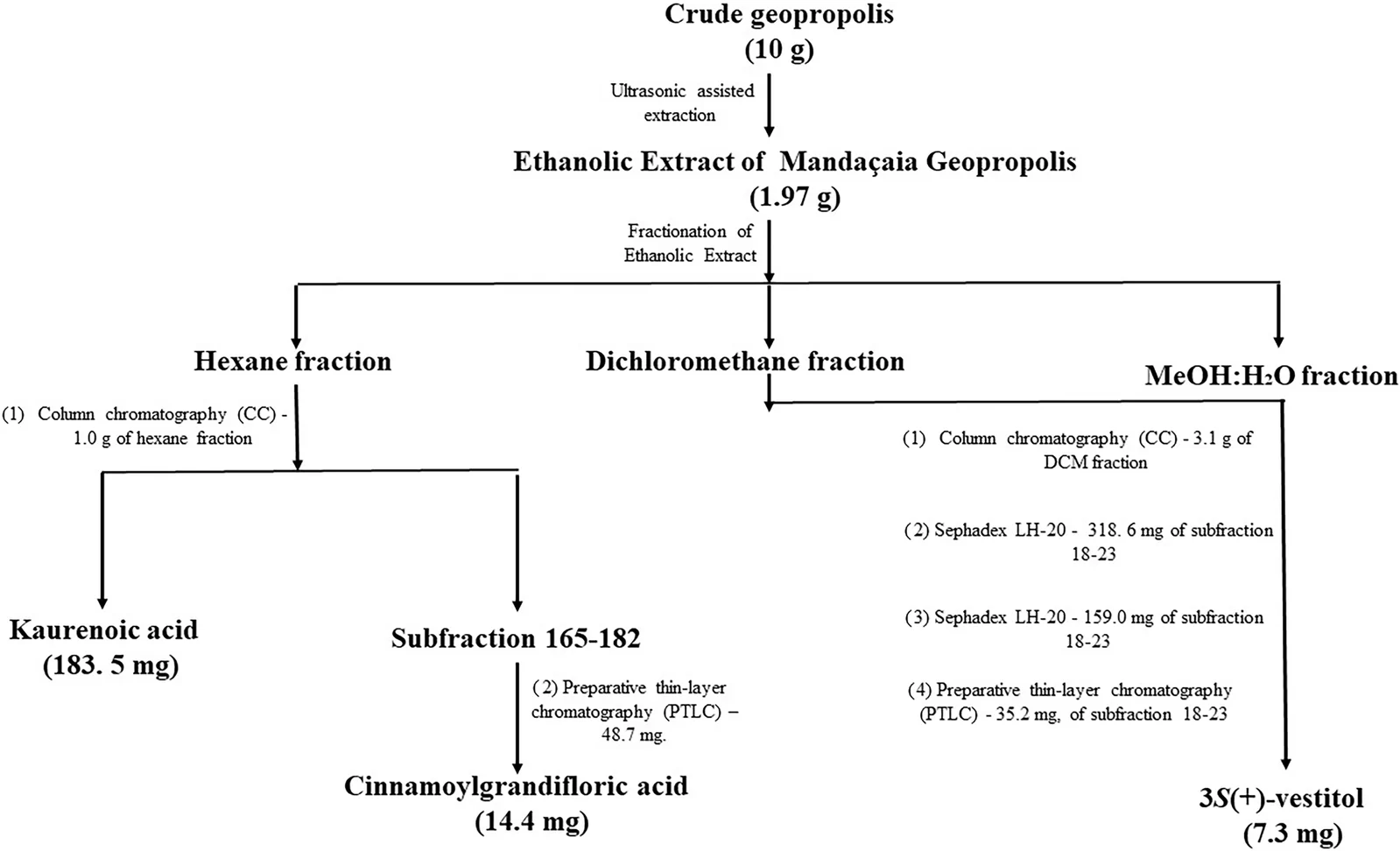
Scheme of Liquid–Liquid Partitioning and Chromatographic Isolation of Compounds from *M. quadrifasciata* *anthidioides* Geopropolis Ethanolic Extract

### 4.4 Total Phenolic Content (TPC)

TPC was determined according to Salgueiro and Castro.^44^ Samples (5 mg mL^–1^ in methanol) were analyzed using the Folin–Ciocalteu method. An aliquot (50 μL) was mixed with diluted Folin–Ciocalteu reagent (1:10, v/v, 2.5 mL) and 4% Na_2_CO_3_ (2.0 mL). After incubation at 50 °C for 5 min, absorbance was measured at 760 nm. Quantification was performed using a gallic acid calibration curve (25–1000 μg mL^–1^), constructed with Origin 6.0 software to yield the linear regression equation *Y* = 0.12497 + 0.12951*X* (*R* = 0.99949), where *Y* represents absorbance and *X* corresponds to gallic acid concentration. The results were expressed as milligrams of gallic acid equivalents per 100 mg of sample (mg GAE 100 mg^–1^). All analyses were conducted in triplicate.

### 4.5 Total Flavonoid Content (TFC)

TFC was determined using the AlCl_3_ method. Samples (5 mg mL^–1^ in methanol) were mixed with 2% AlCl_3_ solution and incubated at 25 °C for 30 min.^44^ Absorbance was recorded at 425 nm. Total flavonoid content was determined using a quercetin calibration curve (1–50 μg mL^–1^). The analytical curve was constructed with Origin 6.0 software to obtain the linear regression equation *Y* = 0.04078 + 0.06553*X* (*R* = 0.99948), where *Y* represents absorbance and *X* corresponds to quercetin concentration. Results were expressed as milligrams of quercetin equivalents per 100 mg of extract (mg QE 100 mg^–1^). All analyses were performed in triplicate.

### 4.6 Evaluation of Antioxidant Capacity by DPPH Free Radical Scavenging Activity Assay

Antioxidant capacity was evaluated using the DPPH free radical scavenging activity assay according to Brand-Williams et al.^45^ with minor modifications. Samples were prepared at concentrations ranging from 50 to 300 μg mL^–1^ in methanol. Aliquots (71 μL) were mixed with 29 μL of freshly prepared DPPH solution (0.3 mmol L^–1^ in methanol) in a 96-well microplate, resulting in a final reaction volume of 100 μL. The mixtures were incubated in the dark at room temperature (25 °C) for 30 min. Absorbance was measured at 517 nm using a Bio-Rad microplate reader. Radical scavenging activity (%) was calculated according to

$$\mathrm{AA}\;\%=100-\frac{\mathrm{AbsA}-\mathrm{AbsB}}{\mathrm{AbsC}}\times 100$$

Where AbsA represents the absorbance of the sample with the DPPH radical solution, AbsB represents the absorbance of the blank for each assay, and AbsC represents the negative control of the assay. EC_50_ values (μg mL^–1^), defined as the concentration required to reduce the initial DPPH absorbance by 50%, were determined by nonlinear regression analysis. All experiments were performed in triplicate.

### 4.7 Isolation of Bioactive Compound

#### 4.7.1 Isolation from the Hexane Fraction

To obtain sufficient material for purification, the crude extract was subjected to successive liquid–liquid partitioning steps. The hexane phases obtained from each partition were combined and concentrated under reduced pressure to afford the accumulated hexane fraction (Fr. Hex). The hexane fraction (Fr. Hex, 1.0 g) was subjected to open-column chromatography on silica gel 60 (230–400 mesh) using a stepwise gradient of hexane–ethyl ether (0–20% ethyl ether). Fractions (100 mL each) were collected and monitored by TLC using vanillin–sulfuric acid reagent for visualization. Similar fractions were combined based on TLC profiles. Subfractions 102–116 afforded **Compound** 1 as a white solid (183.5 mg) after solvent removal. The structure was established by ^1^H and ^13^C NMR spectroscopy and HRMS analysis. Subfractions 165–182 showed multiple components. The UV-active compound (254 nm) was further purified by preparative thin-layer chromatography (PTLC) on silica gel plates (20 × 20 cm^2^; Silicycle, 5–15 μm particle size, 60 Å pore size, with gypsum binder and F_254_ indicator) using appropriate solvent systems, yielding **Compound 2** as a white solid (14.4 mg). Structural elucidation was performed by ^1^H and ^13^C NMR and HRMS.

#### 4.7.2 Isolation from the Dichloromethane Fraction

To obtain sufficient material for purification, the crude extract was subjected to liquid–liquid partitioning in successive batches, and the dichloromethane phases were combined and concentrated under reduced pressure to afford the accumulated dichloromethane fraction. The dichloromethane fraction (3.1 g) was subjected to silica gel column chromatography using a CHCl_3_–MeOH gradient (1–10% MeOH). Forty-seven fractions were collected and pooled according to TLC analysis. Subfraction 18–23 (318.6 mg) was further purified by Sephadex LH-20 chromatography using CHCl_3_–MeOH mixtures. A second LH-20 purification step afforded a subfraction (35.2 mg) that was finally purified by PTLC using toluene–methanol (9:1) to yield **Compound 3** as a white solid (7.3 mg). All isolated compounds were characterized by ^1^H and ^13^C NMR spectroscopy and HRMS.

### 4.8 Antifungal Activity

Antifungal activity was evaluated using the broth microdilution method according to CLSI documents M38-A2 and M27-A3, with modifications for natural products.^46^ Assays were performed in 96-well U-bottom microtiter plates. The culture medium used was RPMI 1640 with l-glutamine, without sodium bicarbonate, and containing phenol red as a pH indicator (Sigma-Aldrich). The medium was prepared according to CLSI recommendations, sterilized by filtration through 0.22 μm membranes under positive pressure, and stored at -8 °C until use. Stock solutions of the extract and itraconazole (positive control) were prepared in dimethyl sulfoxide (DMSO) (Merck, Darmstadt, Germany). Tween 80 was added to the extracts and Compounds 1 and 2 to improve solubility in the culture medium. Serial two-fold dilutions were prepared to obtain final concentrations ranging from 1125 μg mL^–1^ for the crude extract and 1.875 mM for pure compounds. Itraconazole was tested at concentrations from 16 to 0.0125 μg mL^–1^. The clinical strain of *S. brasiliensis* (SisGen ADE44FF) was cultured on Sabouraud Dextrose Agar (KASVI, São José dos Pinhais, Brazil) at 28 °C for 7 days. Conidia were harvested in sterile saline, allowed to decant, and the supernatant was homogenized by vortexing for 15 s. The inoculum was adjusted to 2.0 McFarland standard before addition to the microplates. Plates were incubated at 37 °C for 48 h. The minimum inhibitory concentration (MIC) was determined visually as the lowest concentration that completely inhibited visible fungal growth compared to the growth control. Inoculum and sterility controls were included. All assays were performed in duplicate and independently repeated on two separate days. All procedures involving fungal cultures were conducted in accordance with institutional biosafety guidelines. The strain was registered in the Brazilian National System for the Management of Genetic Heritage (SisGen).

### 4.9 Statistical Analysis

Statistical analyses were performed using GraphPad Prism 8 software (GraphPad Software, San Diego, CA). Quantitative data were expressed as mean ± standard deviation (SD). Data were analyzed by one-way analysis of variance (ANOVA) followed by Tukey’s multiple comparison test. Differences were considered statistically significant at *p* < 0.05. Different superscript letters indicate statistically significant differences between groups.

## Supplementary Material



## Notes

1H NMR, ^13^C NMR, FTIR and LC-MS data for all isolated compounds (PDF)
