# Supplementary material for: Bioactive Constituents of Melipona quadrifasciata anthidioides Geopropolis with Antifungal Activity against Sporothrix brasiliensis
Source: ACS Omega. 2026 Sep 12;11(38):57498–506. doi: 10.1021/acsomega.6c05995 (PMC13628983; doi:10.1021/acsomega.6c05995)
Supplement: Supplementary file 1 [file ao6c05995_si_001.pdf]

Supporting Information
for

Bioactive Constituents of *Melipona quadrifasciata anthidioides* Geopropolis with Antifungal Activity against *Sporothrix brasiliensis*

Gabriel F. dos Santos[a], Maria Clara N. Sampaio[a], Rafael F. dos Santos[a], Aguida A.de Oliveira[b], Rosane N. Castro[a*]

[a] Departamento de Química Orgânica, Instituto de Química, Universidade Federal Rural do Rio de Janeiro, Seropédica, Rio de Janeiro, 23897-000, Brazil.

[b] Departamento de Microbiologia e Imunologia Veterinária, Instituto de Veterinária, Universidade Federal Rural do Rio de Janeiro, Seropédica, Rio e Janeiro, 23890-000, Brazil.

‡These authors contributed equally to this work.

*Corresponding author: nora@ufrrj.br

INDEX:

Figure S1: HPLC-DAD chromatogram (205 nm) of kaurenoic acid. Purity: 99.31% (peak area integration).

Figure S2: FTIR-ATR spectrum of kaurenoic acid.

Figure S3: ^{1}H NMR spectrum (500 MHz, DMSO-d_6) of kaurenoic acid.

Figure S4: ^{13}C NMR DEPT-Q spectrum (125 MHz, DMSO-d_6) of kaurenoic acid.

Table S1: ^{1}H NMR (500 MHz, DMSO-d_6) and ^{13}C NMR (125 MHz, DMSO-d_6) spectrometric data for kaurenoic acid.

Figure S5: Two-dimensional ^{1}H–^{13}C HMBC NMR spectrum (x-axis: δ ^{1}H; y-axis: δ ^{13}C) of kaurenoic acid, showing key long-range correlations used for structural assignment.

Figure S6: HMRS-TOF (MS^{-}) of kaurenoic acid.

Figure S7: HPLC-DAD chromatogram (275 nm) of Cinnamoylgrandifloric acid. Purity: 96.53% (peak area integration).

Figure S8: FTIR-ATR spectrum of cinnamoylgrandifloric acid.

Figure S9: ^{1}H NMR spectrum (500 MHz, DMSO-d_6) of cinnamoylgrandifloric acid.

Figure S10: ^{13}C NMR DEPT-Q spectrum (125 MHz, DMSO-d_6) of cinnamoylgrandifloric acid.

Table S2: ^{1}H NMR (500 MHz, DMSO-d_6) and $1^{3}C$ NMR (125 MHz, DMSO-d_6) spectrometric data for cinnamoylgrandifloric acid.

Figure S11: Two-dimensional ^{1}H–^{13}C HMBC NMR spectrum (x-axis: δ ^{1}H; y-axis: δ ^{13}C) of cinnamoylgrandifloric acid , showing key long-range correlations used for structural assignment.

Figure S12: HMRS-TOF (MS^{-}) of cinnamoylgrandifloric acid.

Figure S13: HPLC-DAD chromatogram (280 nm) of *3S* (+) vestitol. Purity: 95.19% (peak area integration).

Figure S14: FTIR-ATR spectrum of 3*S* (+)-vestitol.

Figure S15: ^{1}H NMR spectrum (500 MHz, $CDCl_3$) of 3*S* (+)-vestitol.

Figure S16: ^{13}C NMR DEPT-Q spectrum (125 MHz, $CDCl_3$) of 3*S* (+)-vestitol.

Table S3: ^{1}H NMR (500 MHz, DMSO-d_6) and $1^{3}C$ NMR (125 MHz, DMSO-d_6) spectrometric data for 3*S* (+)-vestitol.

Figure S17: LC-MS (ESI-) of 3 *S*(+)-vestitol.

Figure S18: Overlaid HPLC-DAD chromatograms (280 nm) of the ethanolic extract (EEGM) and its fractions (Hex, DCM, MeOH:H_2O).

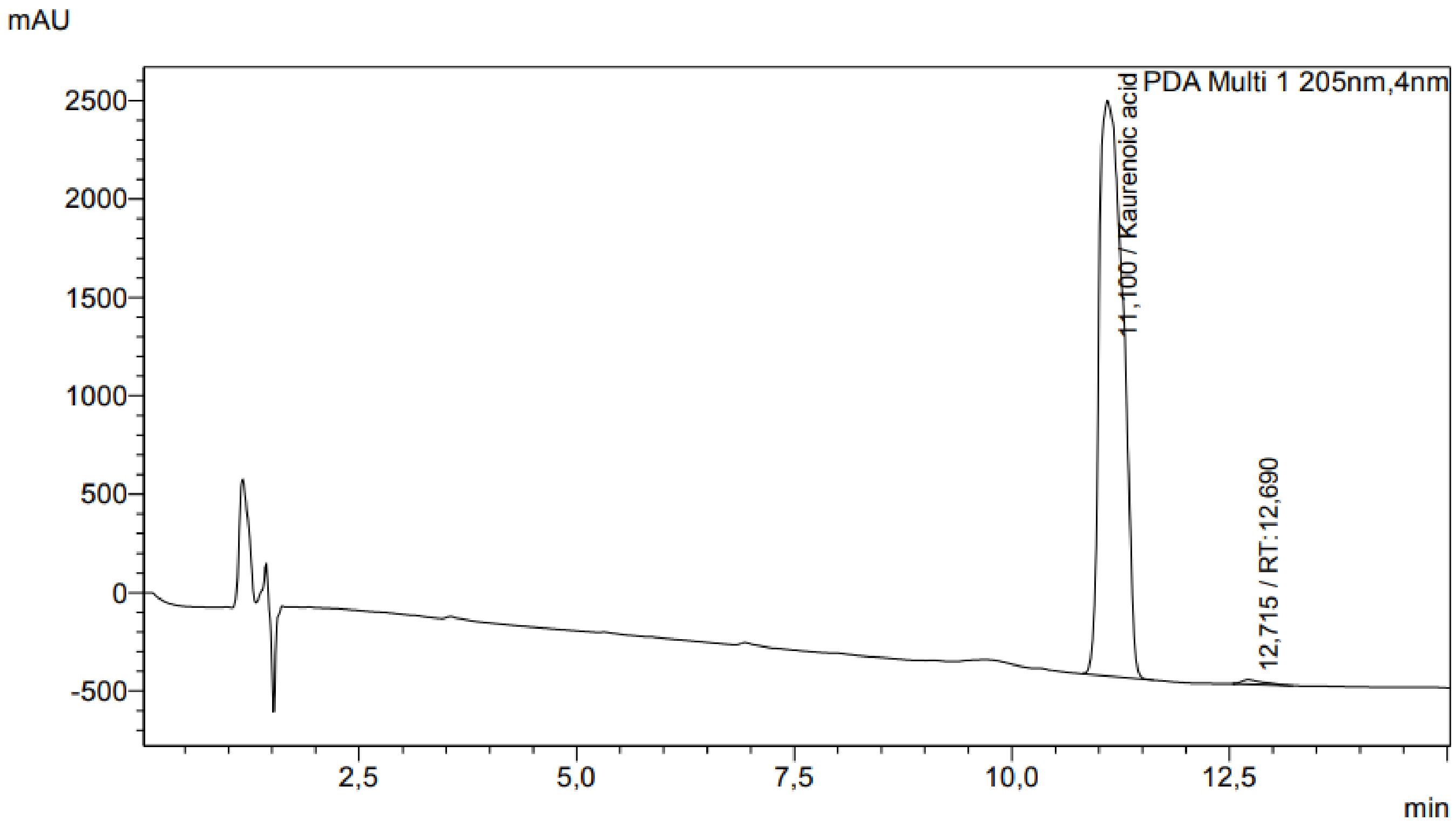

Peak Table

PDA Ch1 205nm

Peak#	Name	Ret. Time	Area%
1	Kaurenoic acid	11,100	99,310
2	RT:12,690	12,715	0,690
Total			100,000

Figure S1: HPLC-DAD chromatogram (205 nm) of kaurenoic acid. Purity: 99.31% (peak area integration).

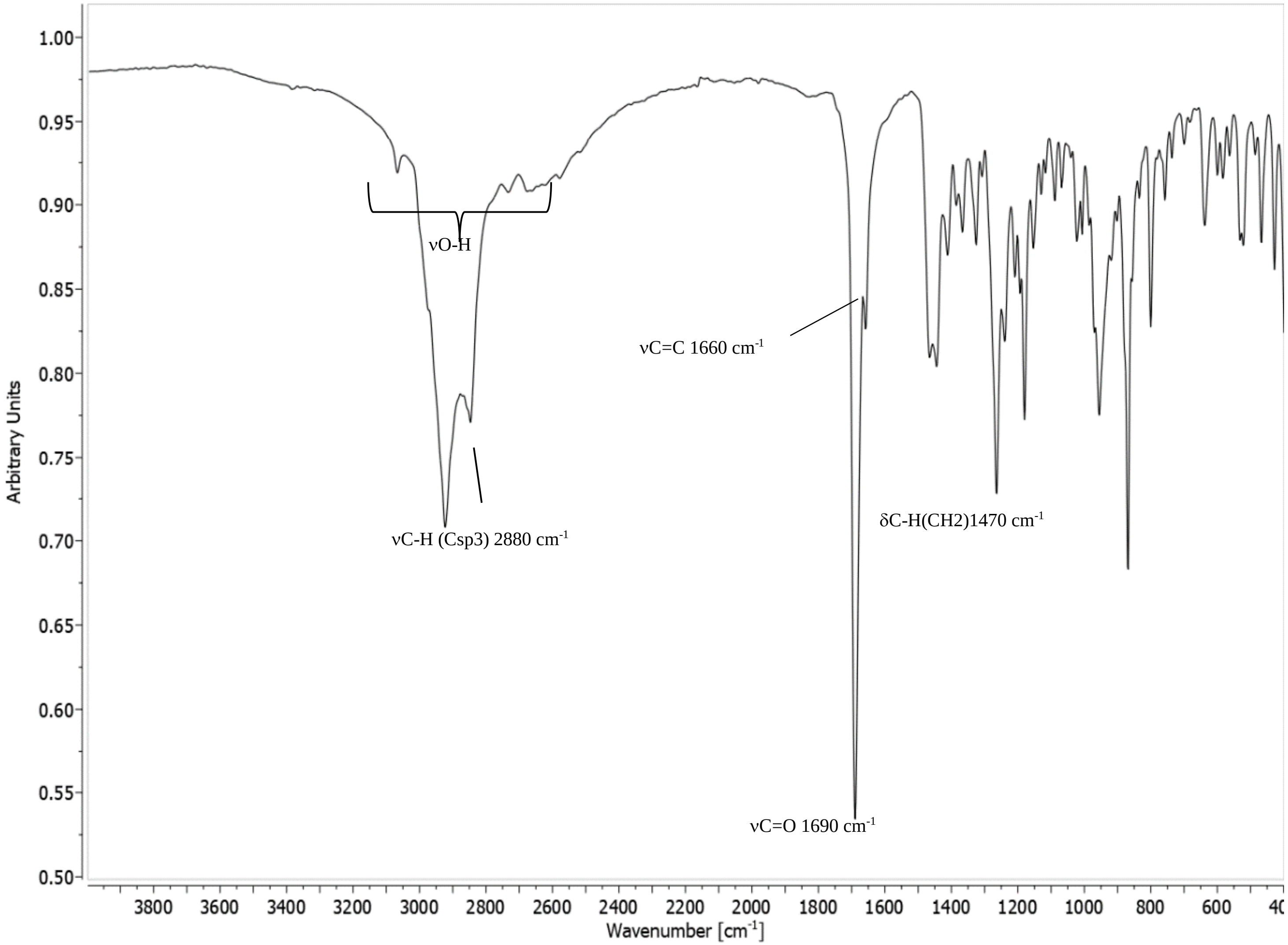

Figure S2: FTIR-ATR spectrum of kaurenoic acid

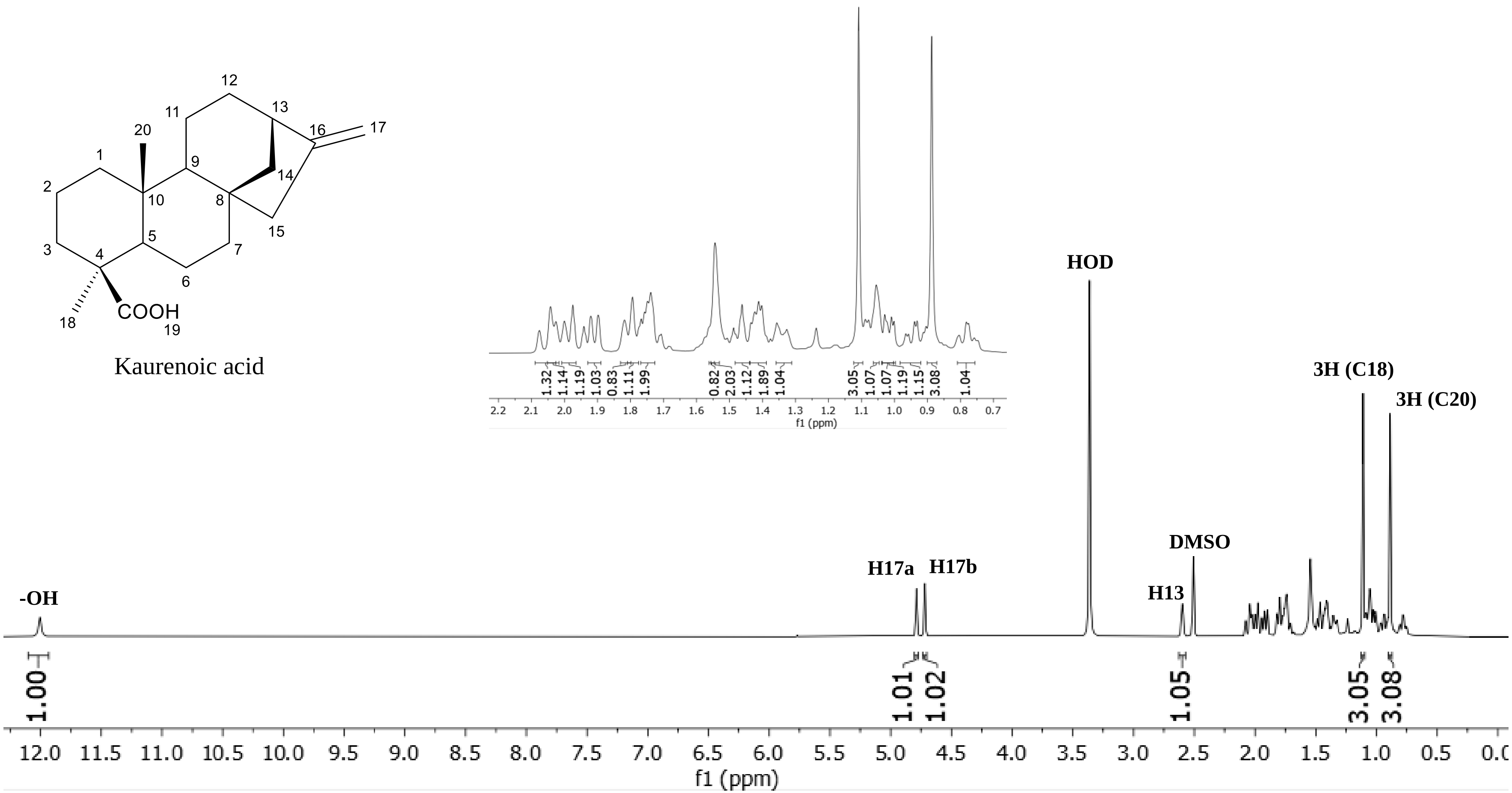

Figure S3: ^1H NMR spectrum (500 MHz, DMSO-d_6) of kaurenoic acid

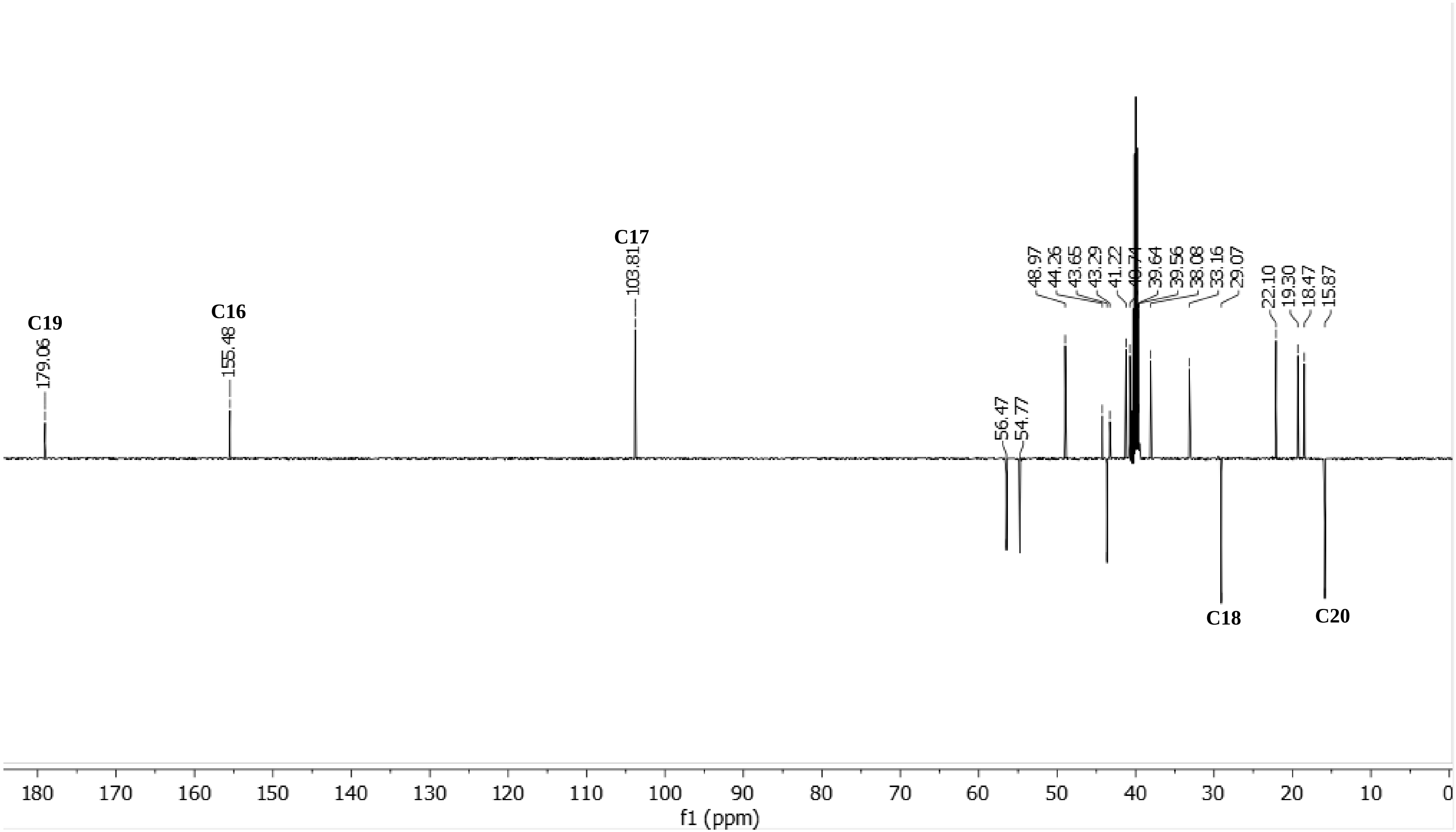

Figure S4: ^{13}C NMR DEPT-Q spectrum (125 MHz, DMSO-d_6) of kaurenoic acid

C	δ_C (ppm)	δ_H (ppm), Multiplicity
4	43.29	-
8	44.26	-
10	39.56	-
16	155.48	-
19	179.06	-
**CH**		
5	56.47	1.02
9	54.77	1.04
13	43.65	2.60, s
**CH_2**		
1	40.70	1.79; 0.78
2	19.30	1.78; 1.35
3	38.08	2.04; 0.94
6	22.10	1.74, m
7	41.22	1.45; 1.39
11	18.47	1.54; 1.55
12	33.16	1.56; 1.41
14	39.64	1.90, d; 1.03
15	48.97	2.05; 1.99
17	103.81	4.78, s; 4.72, s
**CH_3**		
18	29.07	1.10, s
20	15.87	0.89, s

s = *singlet*; d = *doublet*; m = *multiplet*. (;) = non-equivalent H.

Table S1: ^{1}H NMR (500 MHz, DMSO-d_6) and $1^{3}C$ NMR (125 MHz, DMSO-d_6) spectrometric data for kaurenoic acid.

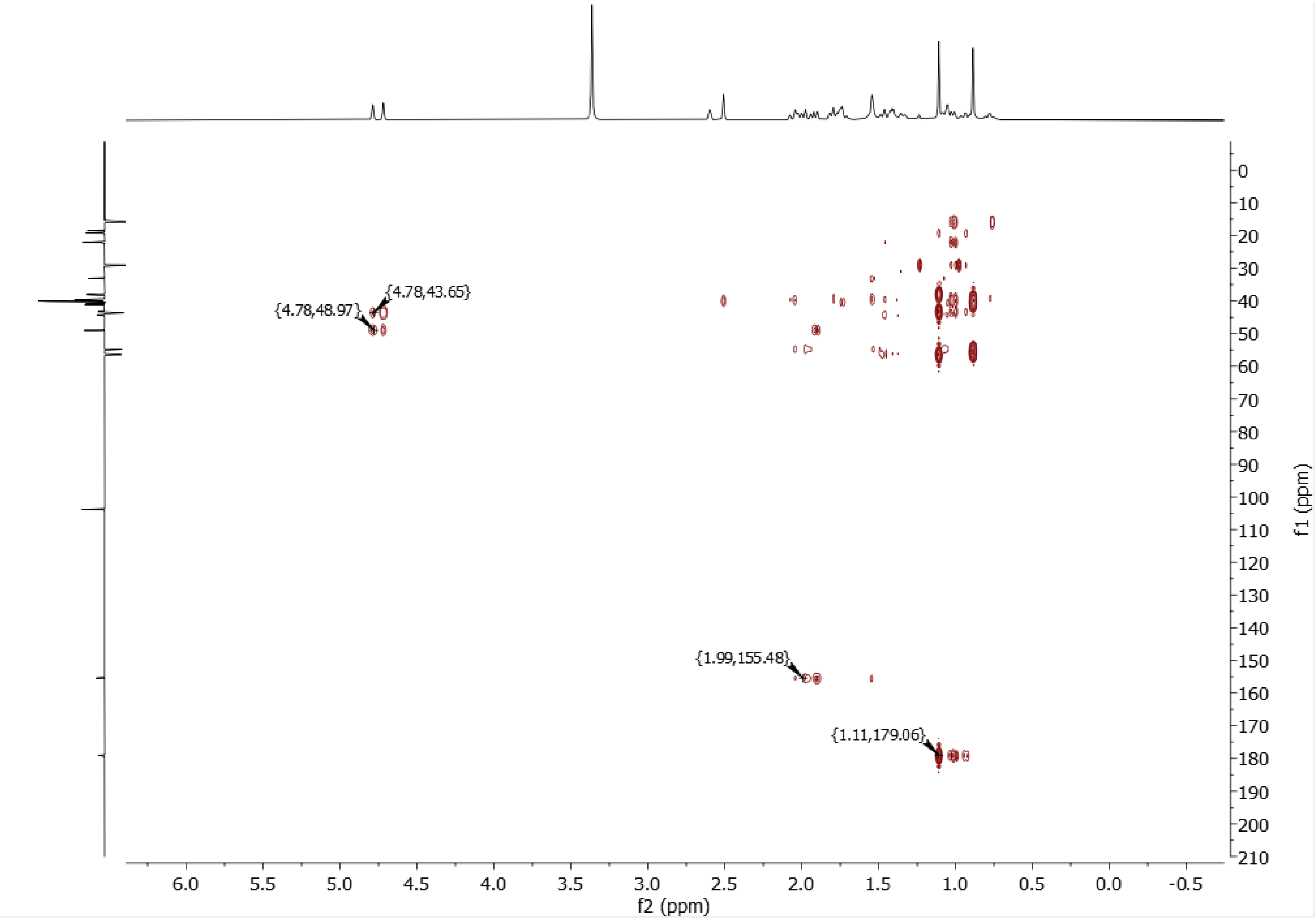

Figure S5: Two-dimensional ^{1}H–^{13}C HMBC NMR spectrum (x-axis: δ ^{1}H; y-axis: δ ^{13}C) of kaurenoic acid, showing key long-range correlations used for structural assignment.

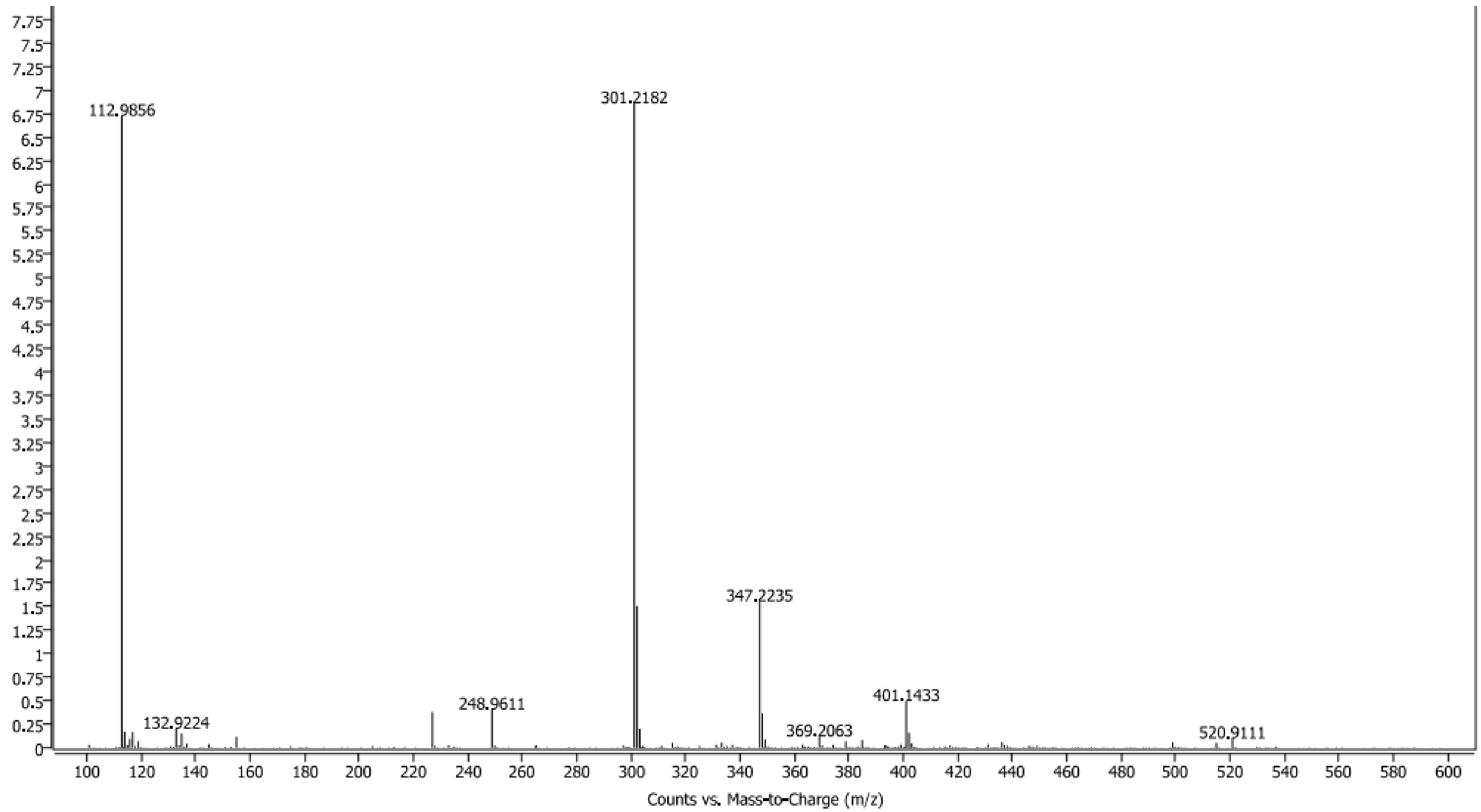

Figure S6: HMRS-TOF (MS^-) of kaurenoic acid.

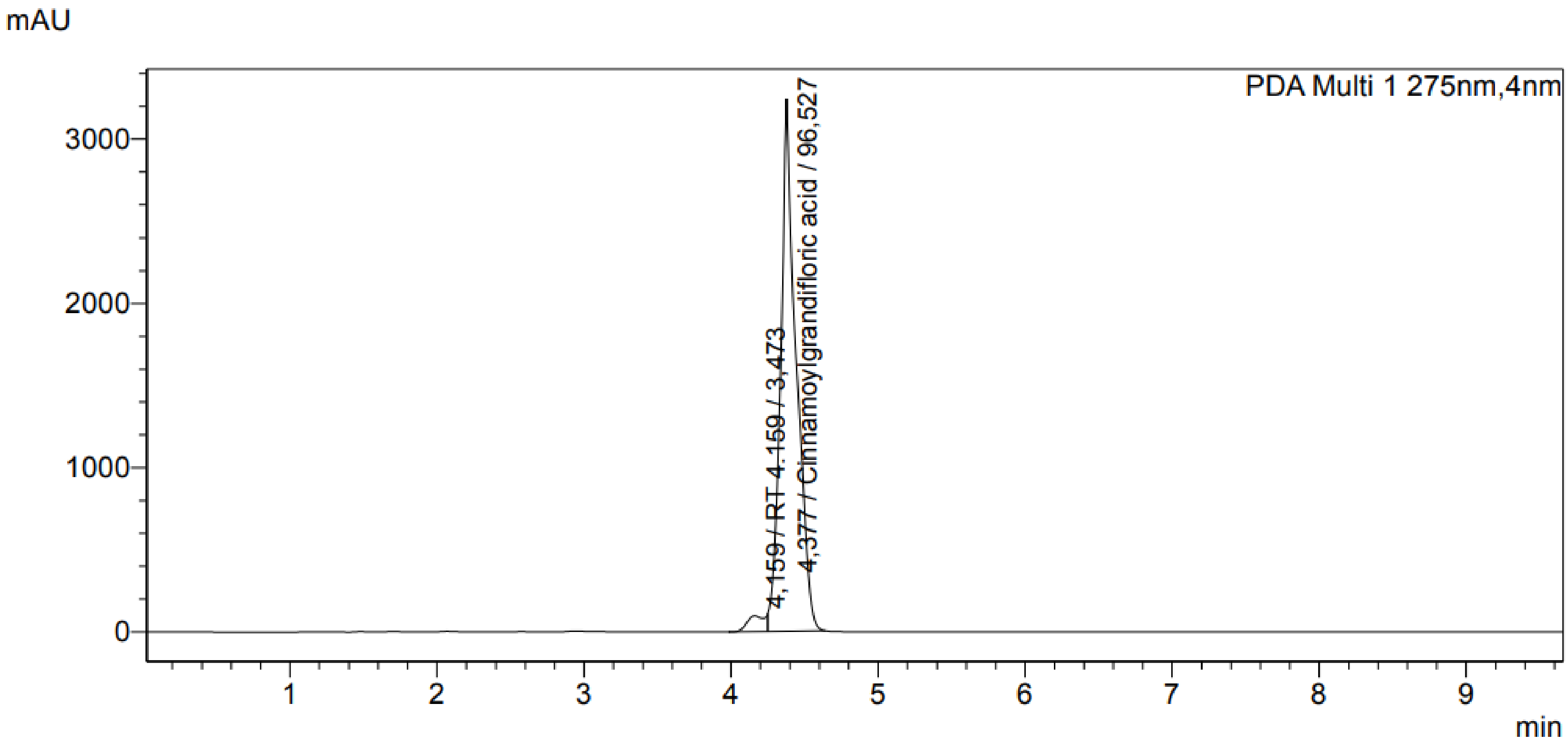

Peak Table

PDA Ch1 275nm

Peak#	Name	Ret. Time	Area%
1	RT 4.159	4,159	3,473
2	Cinnamoylgrandifloric acid	4,377	96,527
Total			100,000

Figure S7: HPLC-DAD chromatogram (275 nm) of Cinnamoylgrandifloric acid. Purity: 96.53% (peak area integration).

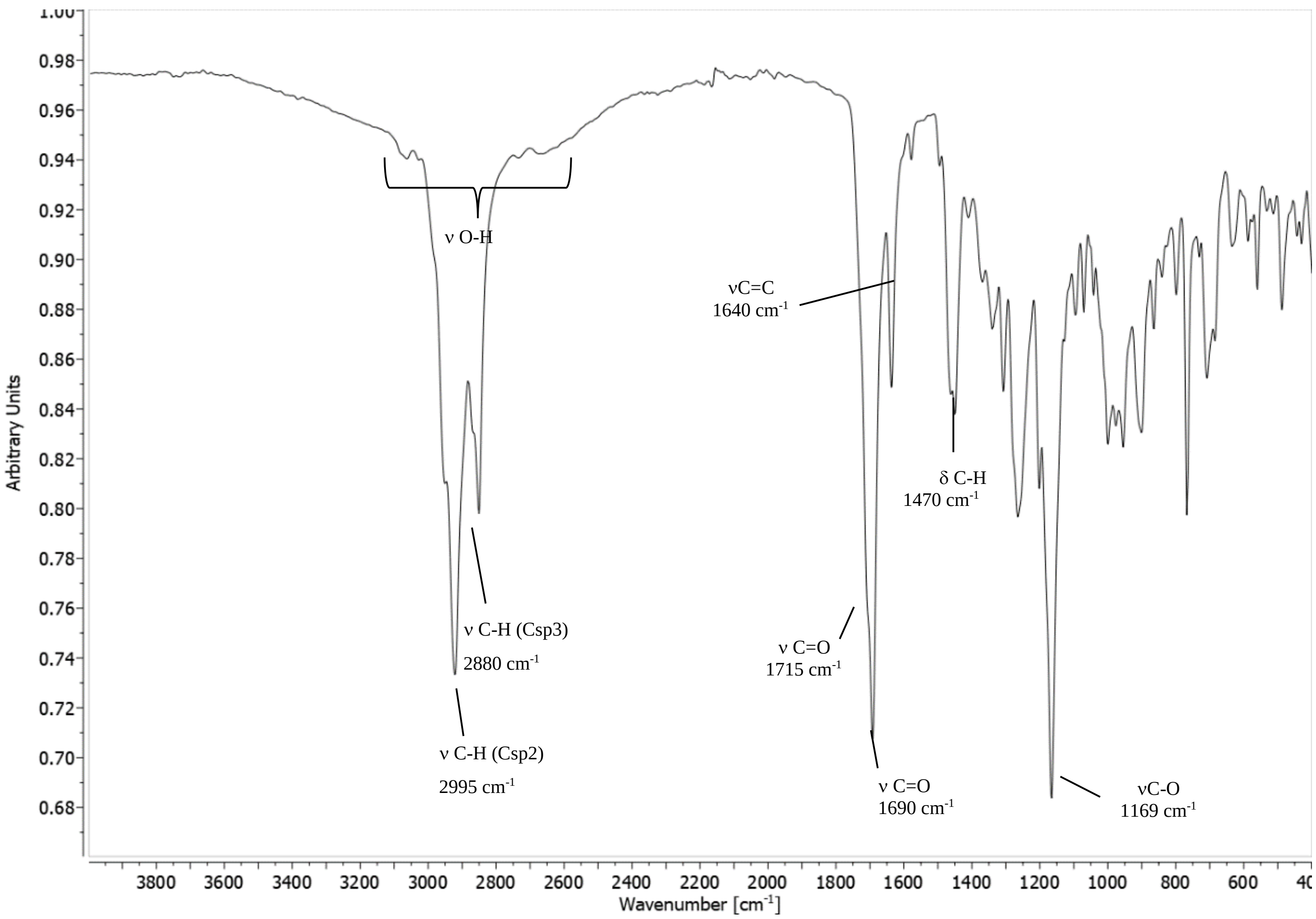

Figure S8: FTIR-ATR spectrum of cinnamoylgrandifloric acid

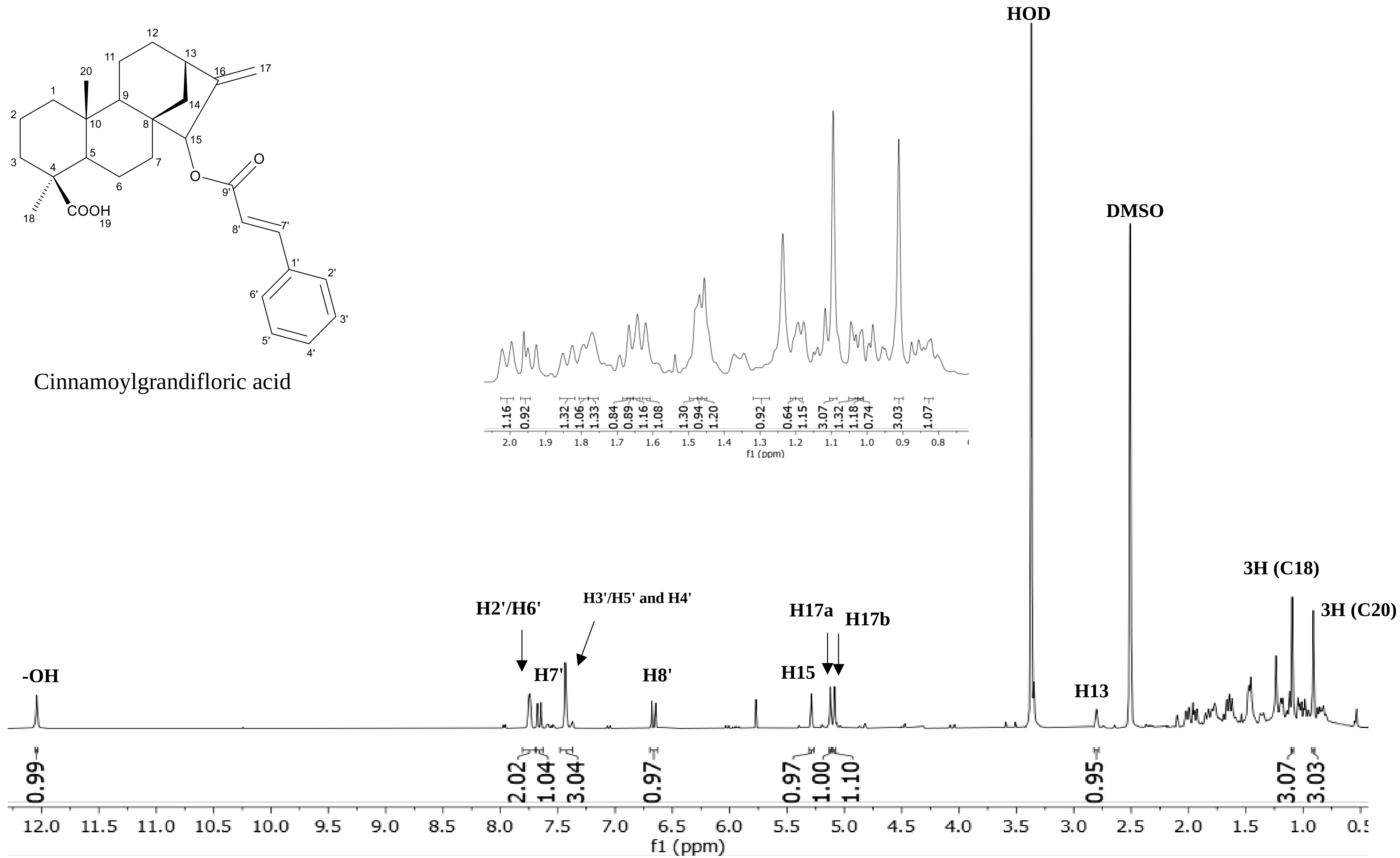

Figure S9: ^{1}H NMR spectrum (500 MHz, DMSO-d_6) of cinnamoylgrandifloric acid

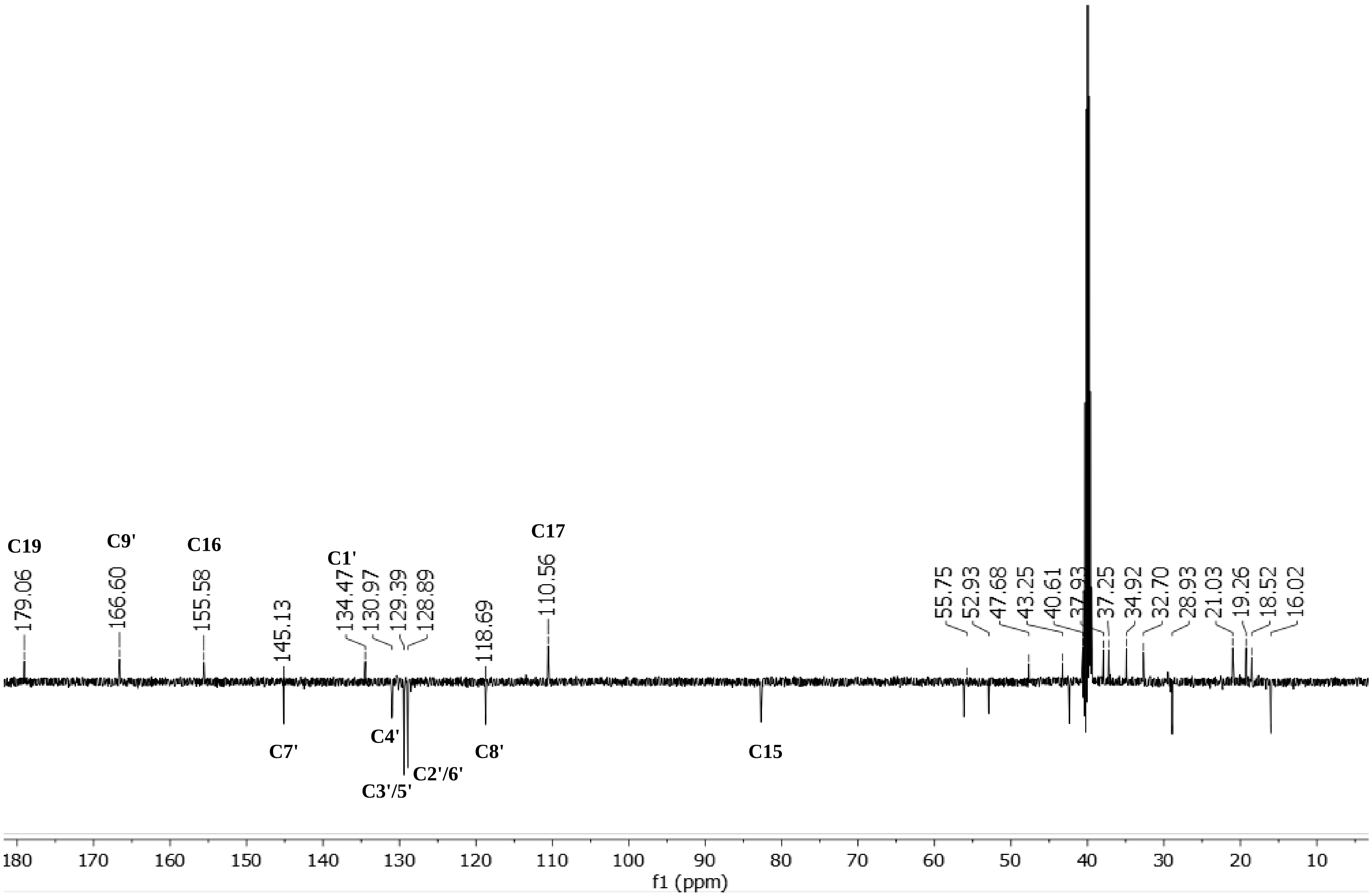

Figure S10: ^{13}C NMR DEPT-Q spectrum (125 MHz, DMSO-d_6) of cinnamoylgrandifloric acid

C	δC (ppm)	δH (ppm), Multiplicity, J (Hz)
4	43.25	-
8	47.68	-
10	40.40	-
16	155.58	-
19	179.06	-
**CH**		
5	55.75	1.03, m
9	52.93	1.19, m
13	42.37	2.80, br s
15	82.72	5.29 s
**CH_2**		
1	40.61	1.82; 0.83
2	19.26	1.78; 1.30
3	37.93	2.01; 0.97
6	21.03	1.79; 1.64
7	34.92	1.65; 1.21
11	18.52	1.62; 1.50
12	32.70	1.63; 1.48
14	37.25	1.95; 1.45
17	110.56	5.10, s; 5.08, s
**CH_3**		
18	28.93	1.01, s
20	16.02	0.95, s
**Cinnamoyl unit**		
1'	134.47	-
2'/ 6'	128.89	7.75, m
3'/5'	129.39	7.43, m
4'	130.97	7.43, m
7'	145.13	7.65, d, 16.0
8'	118.69	6.66, d, 16.0
9'	166.60	-

s = *singlet*; br s = *broad singlet*; d = *doublet*; m = *multiplet*. (;) = non-equivalent H.

Table S2: ^{1}H NMR (500 MHz, DMSO-d_6) and $1^{3}C$ NMR (125 MHz, DMSO-d_6) spectrometric data for cinnamoylgrandifloric acid.

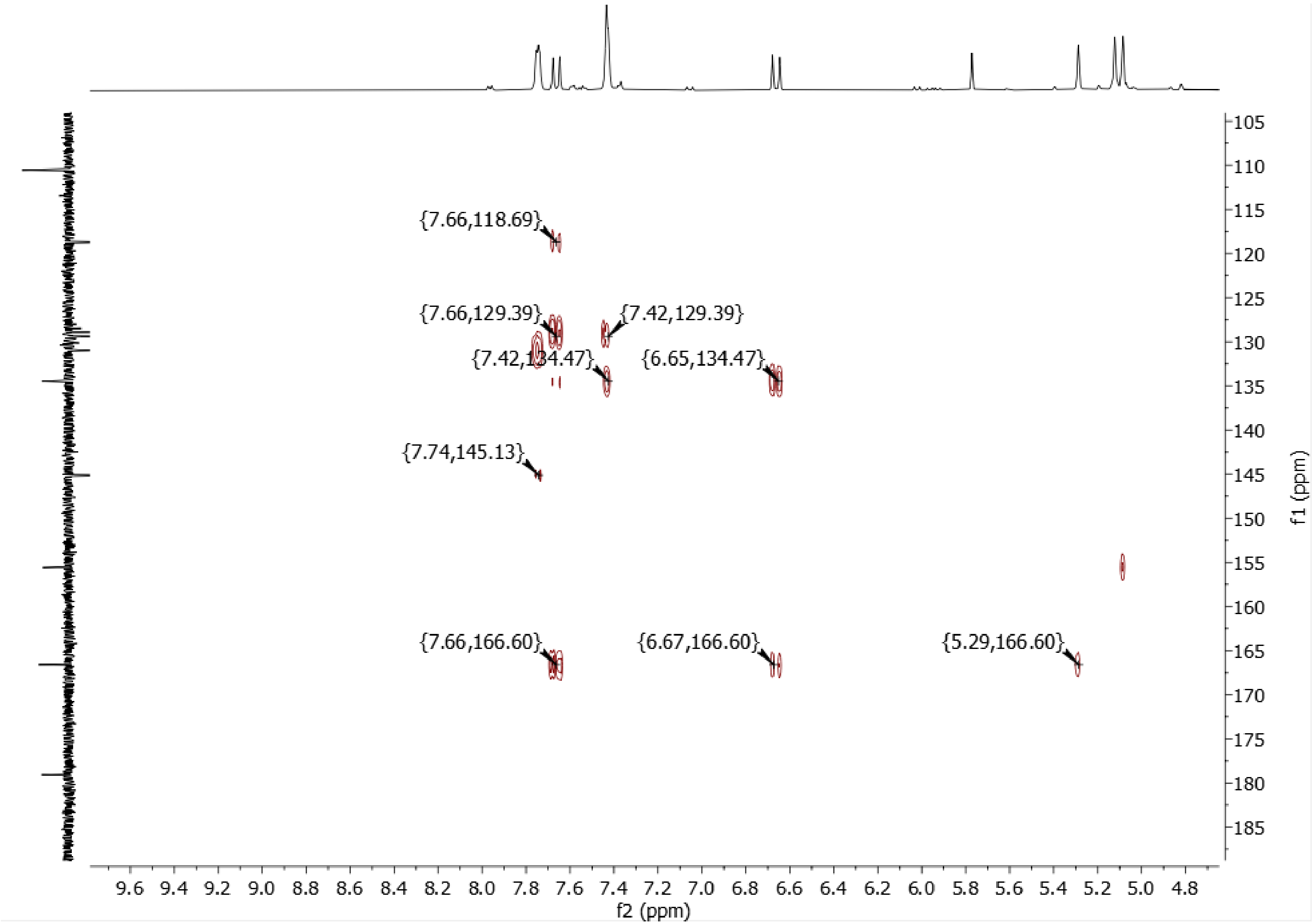

Figure S11: Two-dimensional ^{1}H–^{13}C HMBC NMR spectrum (x-axis: δ ^{1}H; y-axis: δ ^{13}C) of cinnamoylgrandifloric acid , showing key long-range correlations used for structural assignment.

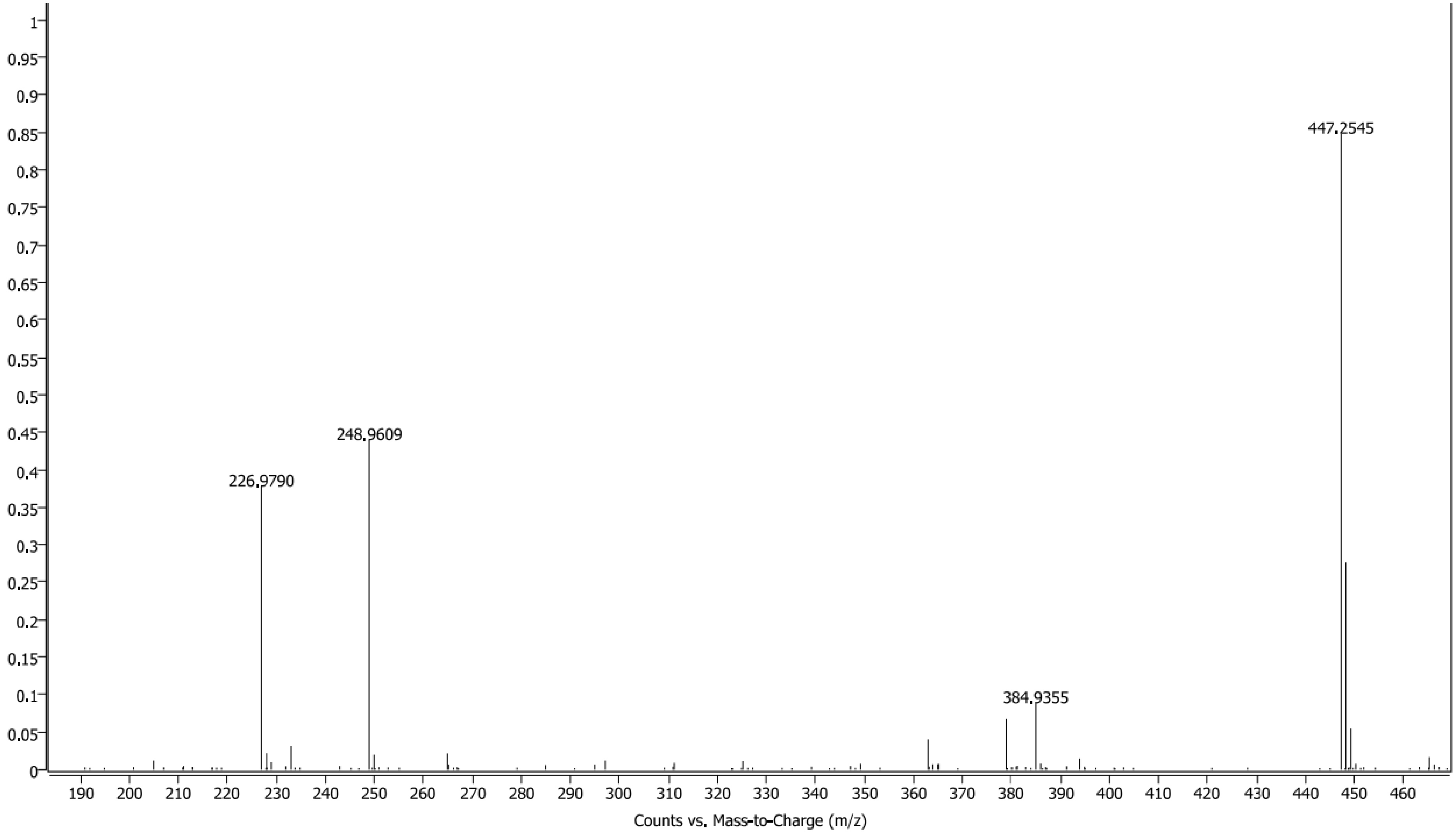

Figure S12: HMRS-TOF (MS⁻) of cinnamoylgrandifloric acid.

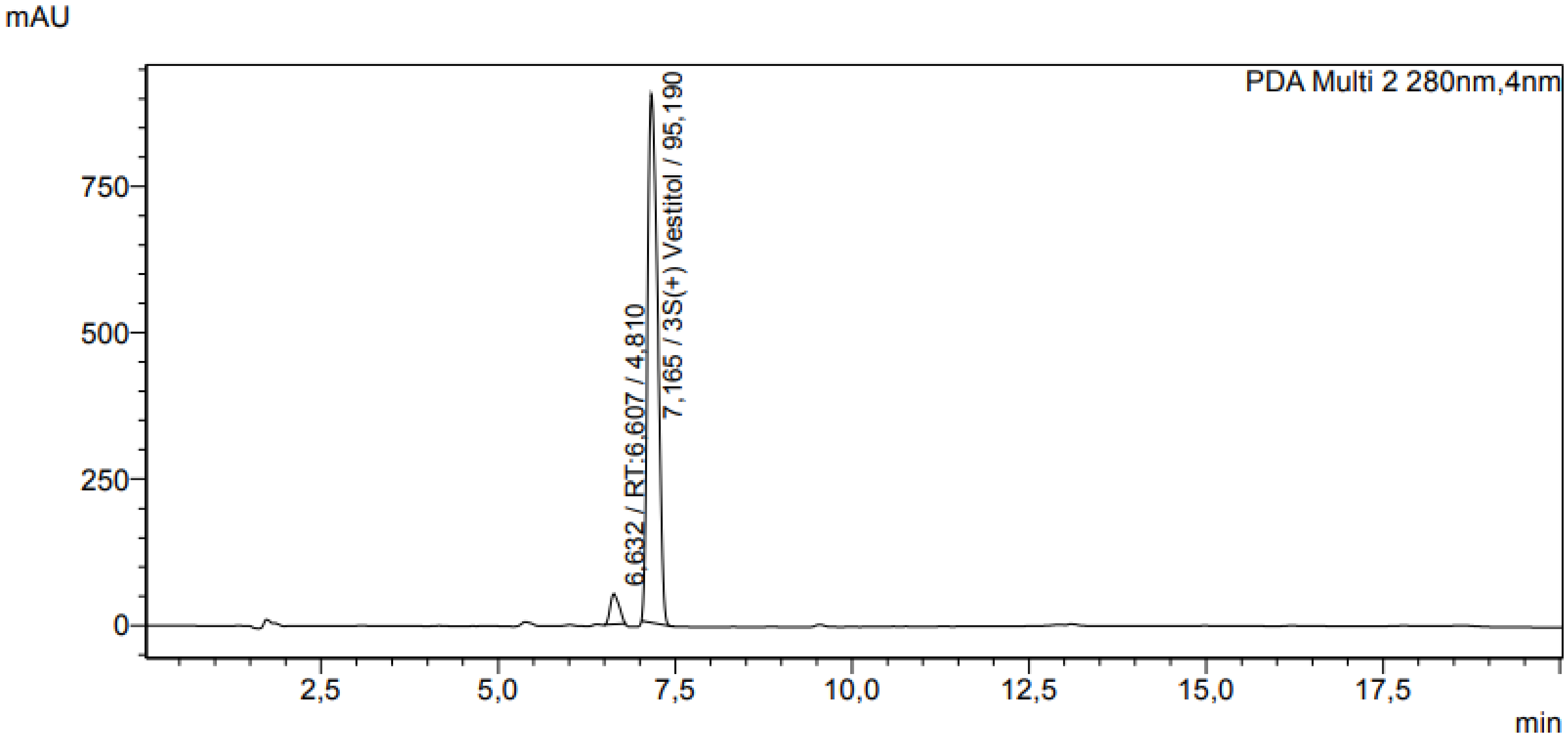

Peak Table

PDA Ch2 280nm

Peak#	Name	Ret. Time	Area%
1	RT:6,607	6,632	4,810
2	3S(+) Vestitol	7,165	95,190
Total			100,000

Figure S13: HPLC-DAD chromatogram (280 nm) of 3*S*(+) vestitol. Purity: 95.19% (peak area integration).

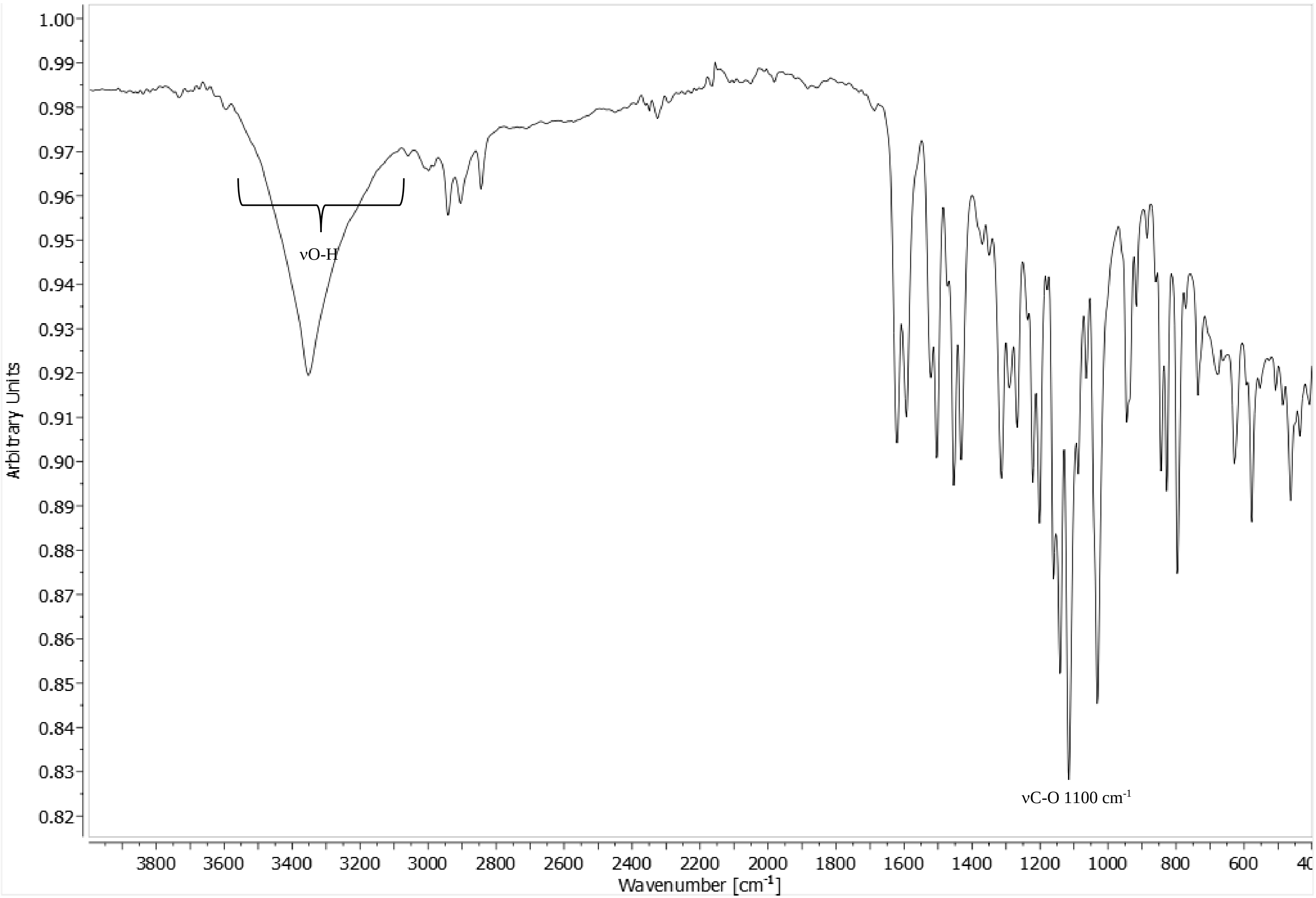

Figure S14: FTIR-ATR spectrum of 3*S*(+)-vestitol

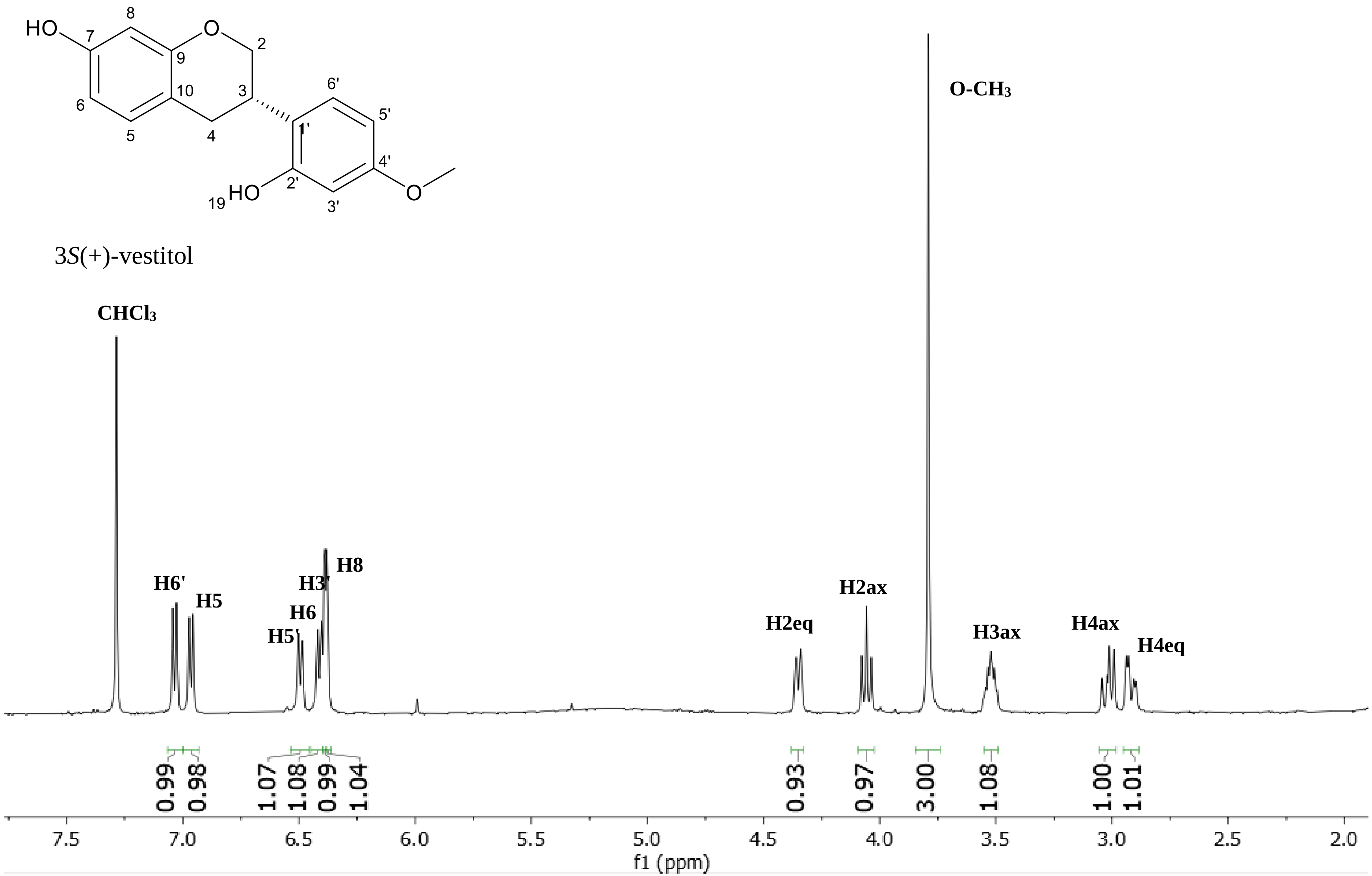

Figure S15: ^{1}H NMR spectrum (500 MHz, $CDCl_3$) of 3*S*(+)-vestitol

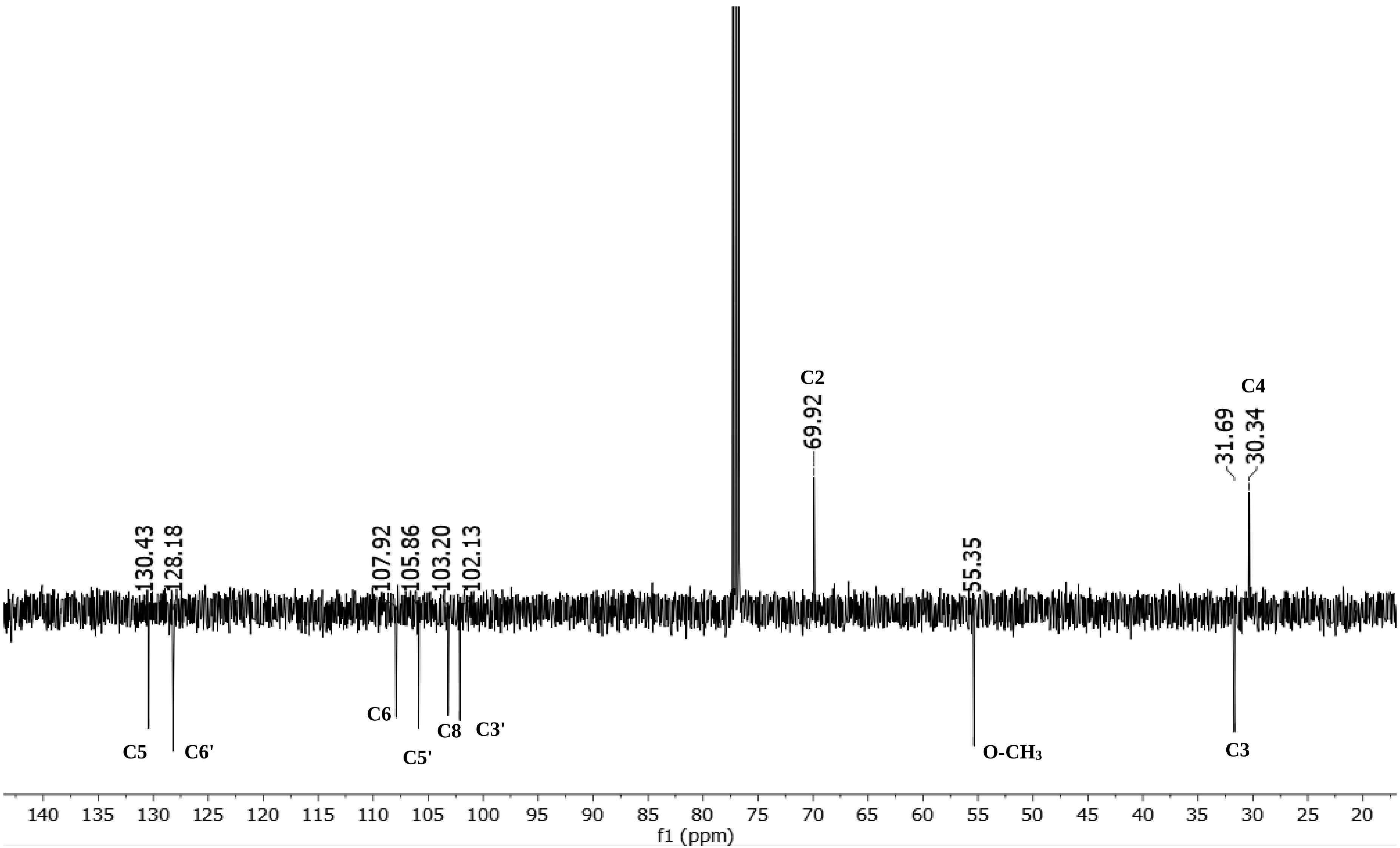

Figure S16: ^{13}C NMR DEPT-Q specrum (125 MHz, $CDCl_3$) of 3*S*(+)-vestitol.

C*	*δC* (ppm)	*δH* (ppm), Multiplicity, *J* (Hz)
7	-	-
9	-	-
10	-	-
**CH**		
3	30.34	3.52, m
5	130.43	6.96, d, 8.1
6	107.92	6.42, dd, 8.6, 2.6
8	103.20	6.38
**CH_2**		
2	69.92	4.36, br d
		4.06, t, 10.2
4	30.34	3.01, m
		2.93, m
**B ring**		
1'*	-	-
2'*	-	-
3'	102.13	6.39
4'*	-	-
5'	105.86	6.51, dd, 8.5, 2.5
6'	128.18	7.04, d, 8.5
O-CH_3	55.35	3.79, s

s = *singlet*; d = *doublet*; dd = *doublet of doublets*; t = *triplet*; br d = *broad doublet*; m = *multiplet*. (;) = non-equivalent H. * Signals for quaternary carbons were not observed under the experimental conditions

Table S3: ^{1}H NMR (500 MHz, DMSO-d_6) and $1^{3}C$ NMR (125 MHz, DMSO-d_6) spectrometric data for 3*S*(+)-vestitol

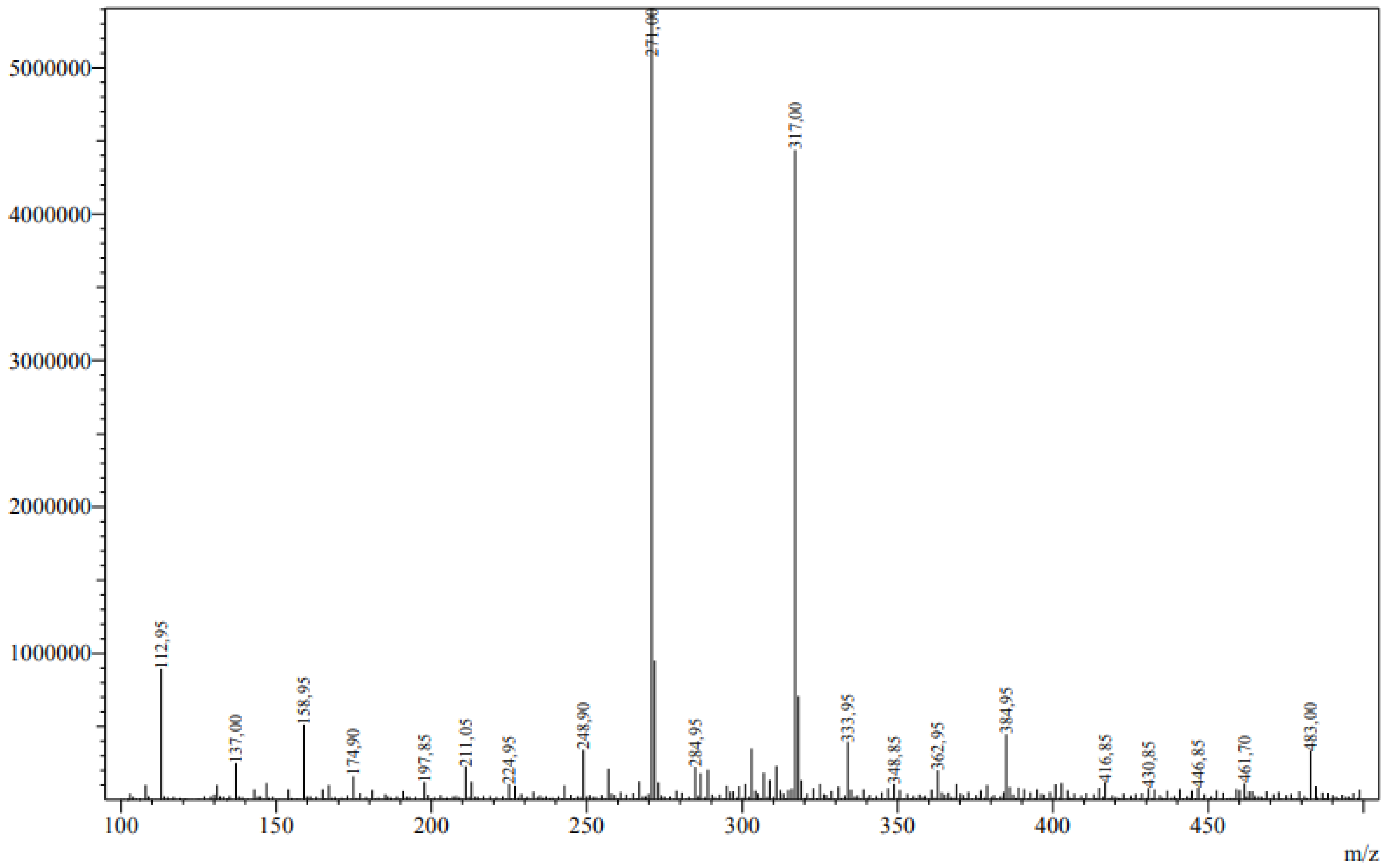

Figure S17: LC-MS (ESI-) of 3*S*(+)-vestitol.

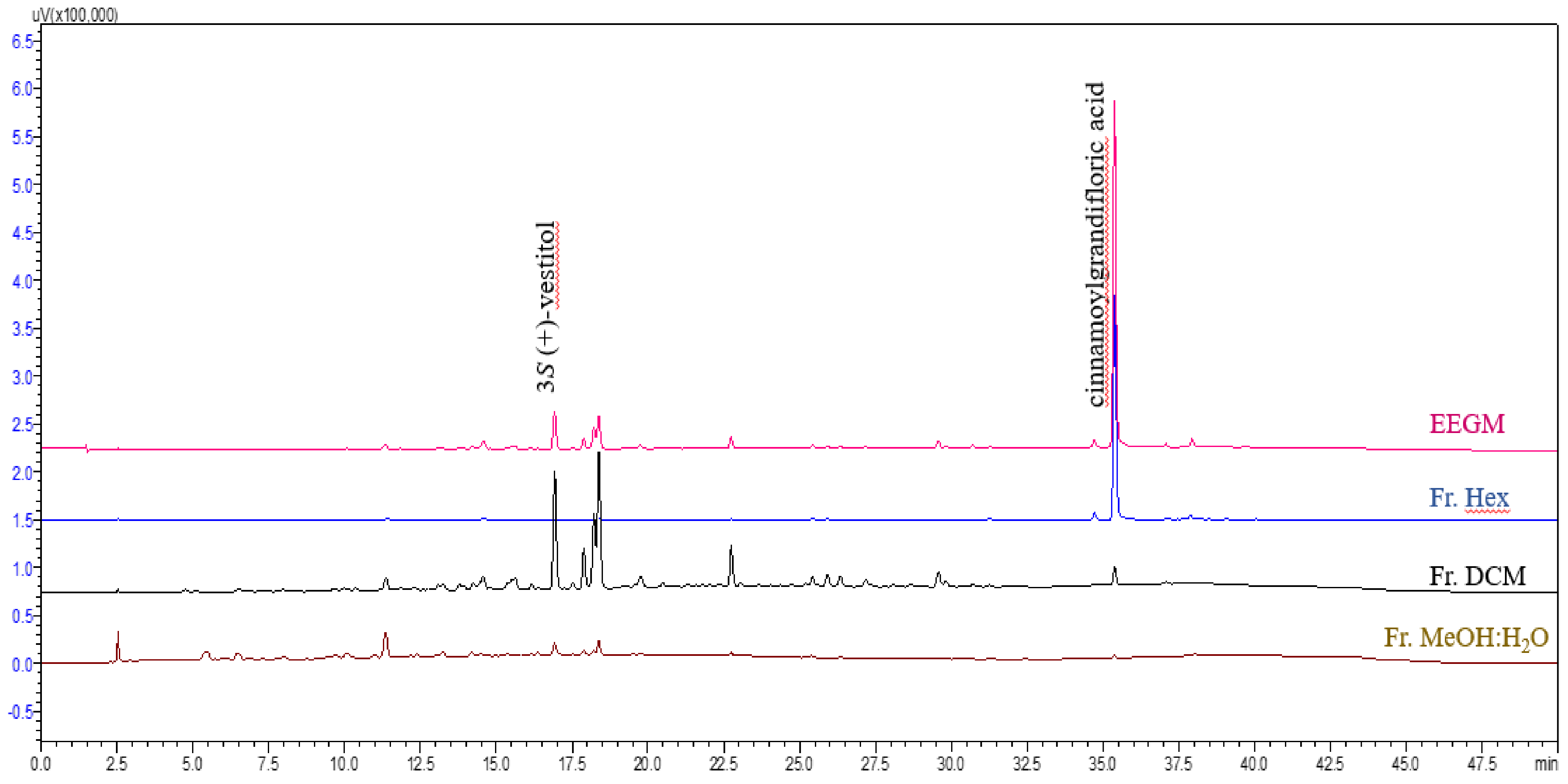

Figure S18: Overlaid HPLC-DAD chromatograms (280 nm) of the ethanolic extract (EEGM) and its fractions (Hex, DCM, $MeOH:H_2O$).
